# Uniparental Markers in Italy Reveal a Sex-Biased Genetic Structure and Different Historical Strata

**DOI:** 10.1371/journal.pone.0065441

**Published:** 2013-05-29

**Authors:** Alessio Boattini, Begoña Martinez-Cruz, Stefania Sarno, Christine Harmant, Antonella Useli, Paula Sanz, Daniele Yang-Yao, Jeremy Manry, Graziella Ciani, Donata Luiselli, Lluis Quintana-Murci, David Comas, Davide Pettener, the Genographic Consortium

**Affiliations:** 1 Laboratorio di Antropologia Molecolare, Dipartimento di Scienze Biologiche, Geologiche e Ambientali, Università di Bologna, Bologna, Italy; 2 Institut de Biologia Evolutiva (CSIC-UPF), Departament de Ciències de la Salut i de la Vida, Universitat Pompeu Fabra, Barcelona, Spain; 3 Institut Pasteur, Human Evolutionary Genetics Unit, Department of Genomes and Genetics, Paris, France; 4 Centre National de la Recherche Scientifique, Paris, France; 5 Dipartimento di Scienze della Natura e del Territorio, Università di Sassari, Sassari, Italy; University of Florence, Italy

## Abstract

Located in the center of the Mediterranean landscape and with an extensive coastal line, the territory of what is today Italy has played an important role in the history of human settlements and movements of Southern Europe and the Mediterranean Basin. Populated since Paleolithic times, the complexity of human movements during the Neolithic, the Metal Ages and the most recent history of the two last millennia (involving the overlapping of different cultural and demic strata) has shaped the pattern of the modern Italian genetic structure. With the aim of disentangling this pattern and understanding which processes more importantly shaped the distribution of diversity, we have analyzed the uniparentally-inherited markers in ∼900 individuals from an extensive sampling across the Italian peninsula, Sardinia and Sicily. Spatial PCAs and DAPCs revealed a sex-biased pattern indicating different demographic histories for males and females. Besides the genetic outlier position of Sardinians, a North West–South East Y-chromosome structure is found in continental Italy. Such structure is in agreement with recent archeological syntheses indicating two independent and parallel processes of Neolithisation. In addition, date estimates pinpoint the importance of the cultural and demographic events during the late Neolithic and Metal Ages. On the other hand, mitochondrial diversity is distributed more homogeneously in agreement with older population events that might be related to the presence of an Italian Refugium during the last glacial period in Europe.

## Introduction

Due to its central position and to the extension of its coastal line (∼7,460 Km), the modern Republic of Italy – e.g. the Italian Peninsula and the two major islands of Sicily and Sardinia – has been one of the focal points in the settlement history of Southern Europe and the Mediterranean Basin.

Populated by early modern humans since approximately 30,000–40,000 years before present (YBP) [1] during the LGM (∼25,000 YBP) it was involved in the southward contraction of human groups from Central Europe that rapidly retreated to the Mediterranean coastlines, occupying refuge areas, such as in the well-known cases of Iberia and the Balkans [2]–[5]. After contributing to the substantial re-shaping of the early Paleolithic genetic composition of glacial Refugia, northward re-peopling processes started approximately 16,000–13,000 YBP [3], [6]–[9].

Subsequently Italy has received the passage of multiple human groups in prehistoric and historic times, acting both as a step point and an area of expansion during the different major migratory events following the early Paleolithic colonization.

The most recent archaeological syntheses [10] describe the early Neolithisation of Italy as the result of two independent and parallel processes, involving respectively the Adriatic and the Tyrrhenian coasts and dating as early as 8,100 YBP (Apulia, South-Eastern Italy) and 7,900 YBP (Liguria, North-Western Italy).

Italian Late Neolithic and the Metal Ages revealed to be a complicated tapestry of different cultural strata, potentially associated with population movements. During the first millennium BC, Italy hosted a vast set of different peoples whose origins in some cases remain unknown (e.g. Etruscans, Ligurians, Veneti), while in other cases are the result of specific migration processes (Celts in North-Western Italy; Greeks in Southern Italy and Sicily) [11].

In addition, independent and/or intersecting subsequent historic events (related with the trade and expansion of different populations in our era: Phoenician, Greek, Carthaginian, Roman, Arabic and Barbaric) also contributed to the present genetic composition of Italy. Unlikely to have completely deleted precedent genetic structures, such migrations may have resulted in partially overlapping patterns of diffusion within Italy.

At present, only few studies addressed the reconstruction of the genetic structure and history of Italian populations. Barbujani and colleagues (1995), in a study based on mtDNA variability [12], identified a North-South gradient within the peninsula, confirming what was previously revealed by classical genetic markers [13], while underlying the genetic differentiation between Sardinia and the mainland [12]. More recent studies focused only on specific regions of Italy and revealed a homogeneous pattern of distribution for mtDNA haplogroups. These findings point towards a substantial homogeneity of the mtDNA gene pool within the different areas of the Peninsula [14], [15].

On the paternal perspective, Di Giacomo et al. (2003) carried out an investigation of Y-chromosome diversity in continental Italy [16]. They identified a single decreasing North-South major cline within the Peninsula, while local drift and founder effects were invoked to explain the observed distribution of genetic variation. The study was replicated by Capelli et al. (2007) with a much larger set of genetic markers and a more specific sampling strategy [17]. They observed that more than 70% of the detected diversity was distributed along latitude-related gradients. A certain level of discontinuity was suggested between Northern and Southern portions of the Italian peninsula that, according to the authors, may be related to differential Neolithic/Mesolithic contributes in the two regions [17]. These results – North-South clinal patterns related to differential Neolithic contributes – were largely confirmed in a recent update of the same study adding more populations and including mtDNA information [18]. Some discontinuity between Northern and Southern Italy was apparent also in genome-wide studies at the European geographical scale [19], [20] and in a specific analysis on Italian samples [21].

Although a common north-south cline has been described for maternal and paternal lineages in Italy, recent data on the Neolithisation of southern Europe [22], [23] suggest a sex-biased Neolithic migration that might account for an asymmetrical pattern of structure in Italy. Eventually more recent migrations could have magnified these sex-biased patterns. For example, this seems to be the case for the first Greek groups in Southern Italy and Sicily, reportedly biased towards a low number of females [11]. Such differential sex-specific demographic events could therefore have affected the genetic structure of Italy in a way that might have been ignored in recent whole-genome analyses.

The present research aims to update our knowledge about Italian population genetic history, by increasing the specificity of sampling strategy and the resolution power of uniparental molecular markers. For the first time, we present an extensive study of both mitochondrial DNA and Y-chromosomal variation in the Italian Peninsula, Sicily and Sardinia. Almost 900 individuals from eight sampling macro-areas have been deeply typed for 136 SNPs and 19 STRs of Y-chromosome, as well as for the whole control region and 39 coding SNPs of mtDNA. We use this detailed and complete dataset to address the following issues. First, we seek to describe the genetic structure of Italy and compare it with the patterns obtained before, in order to distinguish between a clinal and a discontinuous pattern of genetic variation. Second, we want to investigate whether the structure observed is sex-biased and which factors could account for any differential contributes from paternal and maternal lineages. Third, we seek to identify which population movements mostly could be in the origin of the current genetic diversity of the Italian populations.

## Materials and Methods

### Ethics Statement

For all subjects, a written informed consent was obtained, and Ethics Committees at the Universitat Pompeu Fabra of Barcelona (Spain), and at the Azienda Ospedaliero-Universitaria Policlinico S.Orsola-Malpighi of Bologna (Italy), approved all procedures.

### Sample collection

A total of 884 unrelated individuals from continental Italy, Sicily and Sardinia were collected according to the following sampling strategy. Firstly, based on the results of a precedent reconstruction of the surname structure of Italy [24], we defined lists of monophyletic surnames for each of the 96 Italian provinces. Secondly, monophyletic surnames frequencies were used to define eight clusters of homogeneous Italian provinces (sampling macro-areas, Figure S1). Within each sampling macro-area, we selected a set of provinces (sampling points) from a minimum of one to a maximum of three, depending on the geographical extension of the macro-area as well as their historical background. This was done in order to depict a sampling grid able to capture as much genetic variability as possible (given the number of planned samples/sampling points). Within each sampling point, individuals were finally sampled according to the standard ‘grandparents’ criterion, thus considering as eligible for our study only those individuals whose four grandparents were born in the same sampling macro-area. It is important to underline that individuals within sampling points were not selected by surnames. That way 1) our data are consistent with those from other similar studies; 2) we avoid to introduce a bias between Y-chromosome and mtDNA results.

DNA was extracted from fresh blood by a Salting Out modified protocol [25].

### Y-chromosome genotyping

A total of 884 samples were successfully typed for Y-chromosome markers. 121 SNPs in the non-recombining region of the Y chromosome were genotyped using the OpenArray® Real-Time PCR System (Applied Biosystems) as described previously [26]. Six additional SNPs (M91, M139, M60, M186, M175, and M17) were genotyped in a single multiplex, Multiplex2 [27]. Nine additional single SNPs (M227, L22, M458, L48, L2, L20, M320, P77) were typed with individual TaqMan assays. Nomenclature of the haplogroups is in accordance with the Y-Chromosome Consortium [28]. Detailed phylogeny may be found at Y-DNA SNP Index - 2009 (http://isogg.org/tree/ISOGG_YDNA_SNP_Index09.html). For simplicity reasons, we will use asterisks (*) to indicate those chromosomes that are derived at a certain SNP, but ancestral at all the tested downstream SNPs.

All individuals were additionally typed for a set of 19 STRs: 17 using the Yfiler kit (Applied Biosystems) and two (DYS388, DYS426) included in the Multiplex2. As the Yfiler kit amplifies DYS385a/b simultaneously avoiding the determination of each of the two alleles (a or b), DYS385a/b were excluded from all the analyses performed. DYS389b was obtained by subtracting DYS389I from DYS389II [29].

### Mitochondrial DNA genotyping

865 samples were successfully sequenced for the whole control region as in Behar et al. (2007) [30], and typed using a 22 coding region SNPs multiplex as described previously [27], [31]. Variable positions throughout the control region were determined between positions 16,001 and 573. Sequences were deposited in the GenBank nucleotide database under accession numbers KC806300-KC807164. In addition, for haplogroup H, the most frequent in Western Europe [2], [6], we used a specifically designed multiplex (named HPLEX17) in order to resolve 17 distinct sub-lineages [27]. Based on combined HVS sequence and coding region SNP data, individuals were assigned to the major haplogroups of the mtDNA phylogeny with the software Haplogrep [32] that uses Phylotree version 13 [33]. Due to their phylogenetic uncertainty, indels at nucleotide positions 309, 315, and 16193 were not taken into account.

### Statistical Analyses

#### Population structure and genetic variability

Haplogroup frequencies were estimated by direct counting. Standard diversity parameters (haplogroup diversity, number of observed STR haplotypes, sequence diversity values, and mean number of pairwise differences) were calculated with Arlequin 3.5 [34]. FST and RST results were corrected with Bonferroni test for multiple comparisons (p<0.05).

The relationships between geographical distances and genetic diversity were investigated by using several spatial analyses. The correlation between geographical distances and genetic distances (Reynolds distance), based on haplogroup frequencies, was evaluated by means of a Mantel test (10,000 replications). In order to distinguish any clinal pattern (Isolation-by-Distance pattern) from any discontinuous genetic structure (both of them can result in significant correlations with geography), geographical distances were plotted against genetic ones. A 2-dimensional kernel density estimation layer [35] was added to the plot in order to highlight the presence of discontinuities in the cloud of points. The analysis was performed with all the samples and then removing the Sardinian ones, given their outlier status previously described in literature [7], [13], [21], [36]–[38].

To further explore spatial patterns of variation a spatial principal component analysis (sPCA) based on haplogroup frequencies was performed using the R software package *adegenet* [39]–[41]. Additional information about the sPCA method is provided in Methods S1.

To further test the significance of the structure found with the sPCA analysis, we carried out a series of hierarchical analyses of molecular variance (AMOVA) pooling populations according to the sPCA results. We used haplogroup frequencies (both Y-chromosome and mtDNA), RST distances (Y-STRs) and number of pairwise differences (HVRI-HVRII mtDNA sequences). In order to explore genetic variability within the most frequent haplogroups, and in particular within those identified by sPCA loadings, we applied a Discriminant Analysis of Principal Components (DAPC) to Y-STR haplotypes and mtDNA sequences (see Methods S1 for more details). Analyses were performed using the R software *adegenet* package [39]–[41]. In addition, for comparison purposes we calculated a Network representation of haplogroup G2a using a Median Joining (MJ) algorithm as implemented in the Network 4.6.1.1 software (http://www.fluxus-engineering.com, [42]), weighting STR loci according to the variance method.

DAPC was first performed using Italian haplotypes only. As a second step, in order to investigate the origin of the genetic diversity for the most common haplogroups in Italy, additional individuals from selected European populations were incorporated into the DAPC of major haplogroups. Unpublished 194 Y-chromosome data from Iberia, Germany and the Balkans were provided by the Genographic Project, while data for Causasus and Western Anatolia were extracted from literature [43], [44]. Comparison data for mtDNA was generated using additional information from Basque [45], Austrian [46] and Balkan samples [46], [47].

#### Y-chromosome and mtDNA dating

In order to minimize the biasing effect of STRs saturation through time (especially important for rapidly evolving STRs as some of those included in the Yfiler kit, [48]), all Y-chromosome age estimations were calculated selecting the eight markers (DYS448, DYS388, DYS392, DYS426, DYS438, DYS390, DYS393, DYS439) with the highest values of duration of linearity D approximated as in Busby et al. (2011) [49].

Splitting time between the sPCA-identified regions (NWI and SEI, see Results) was estimated with BATWING [50] under a model of exponential growth and splitting from a constant size ancestral population. Two samples (Treviso, Foligno/PG) were excluded from the analysis according to a 5% quantile threshold of the sPC1 scores. Two chains with different starting points were run with a total of 3.5×10[6] samples with an initial burn in of 1.5×10[6] samples and a thinning interval of 10×20. The outfiles were treated with the R package [41] to get the posterior distributions of the parameters of interest. We checked that results were equivalent for both runs and reported the mean values of both analyses for every parameter. We used a prior distribution for mutation rates as proposed by Xue et al. (2006) [51] based on Zhivotovsky et al. (2004) [52]. Such distribution is wide enough to encompass all mutation rates for each of the eight considered Y-STRs. A generation time of 25 years was used [52]. Priors and further information about the BATWING procedure are shown in the Methods S1.

The age of Y-chromosome DAPC clusters exhibiting peaks of frequency higher than 70% in any of the sPCA-identified populations (NWI, SEI, and SAR) – with the exception of haplogroup G2a due to its particular relevance in our populations (see Results) – and composed by at least ten individuals, as well as the age of the entire haplogroups, were estimated with the standard deviation (SD) estimator [53]. Differently from BATWING, this method does not estimate the population split time, but the amount of time needed to evolve the observed STRs variation within haplotype clusters (or whole haplogroups) at each population. As for mutation rates, we adopted locus-specific rates for each of the eight considered loci as estimated by Ballantyne et al. (2010) [48]. These rates were preferred to the ‘evolutionary’ one [52] for the following reasons: 1) ‘germline’ rates are locus-specific and based on the direct observation of transmission between father-son pairs; 2) ‘germline’ rates share the same magnitude with genealogy based estimates [54] while the ‘evolutionary’ rate is a magnitude lower; 3) a recent study [43] suggested that family based rates (germline, genealogies) provide a better fit with history and linguistics. The 95% confidence intervals of time estimates were calculated based on the standard error (SE). Only individuals with a membership >99% in their corresponding DAPC clusters were considered. Given that moments like mean and variance – hence time estimates based on variance – are very sensitive to the presence of outliers (e.g. non-robust), we designed a “jackknife-like” procedure in order to detect possible outlier individuals that could be significantly biasing our estimates (see Methods S1 for details).

TMRCA for the most common mtDNA haplogroups was estimated by means of the ρ (rho) statistic with the calculator proposed by Soares et al. (2009) [55] for the entire control region (that considers a mutation rate corrected for purifying selection of one mutation every 9,058 years).

However, results have to be taken with caution, given that molecular date estimates with ρ can be affected by past demography. Simulations show that error rates tend to increase with effective size, bottleneck and growth effects [56]. In order to avoid sampling errors, the estimates were calculated only for those haplogroups with absolute frequencies higher than 30 individuals.

## Results

### Y-chromosome lineages in Italy

#### Haplogroup frequencies

A total of 884 unrelated individuals from 23 Italian locations (Figure S1) were successfully genotyped for 19 STRs and 136 SNPs, and classified in 46 different haplogroups (including sub-lineages) whose phylogeny ([28]; ISOGG Y-DNA SNP Index – 2009) and frequencies for the whole dataset are detailed in Table S1; Y-STR haplotypes of each individual are provided in Table S2.

The haplotype and haplogroup diversity (*h*), STR diversity (*π_n_*) and mean number of pairwise differences (*π*) of the population samples are listed in Table S3. The lowest values for haplogroup diversity (*h*) are observed in Sardinia, while the Italian peninsula is characterized by a negative correlation between haplogroup diversity and latitude, resulting in a south-north decreasing pattern of variation (Spearman's rho  = −0.463, p-value  = 0.036). The most frequent haplogroups in Italy are R-U152* (12.1%), G-P15 (11.1%), E-V13 (7.8%) and J-M410* (7.6%). They are followed by three R1b-lineages (R-M269*, R-P312* and R-L2*), whose frequencies ranged from 6.9% to 5.7%; and finally from I-M26, which embraced more than the 4% of total variability. On the whole these haplogroups encompass ∼62% of Y-chromosomes lineages, while the remaining 38 haplogroups show frequencies lower or equal to 3.3%. Haplogroups distribution in the considered eight sampling areas is detailed in Table S1.

#### Paternal population structure

In order to explore the relationship between geographical and paternal genetic distances among the 23 investigated Italian populations a Mantel test was performed. A significant correlation was found (observed value  = 0.26, p-value  = 0.006), even after removing Sardinian samples (observed value  = 0.19, p-value  = 0.03). However, a non-homogeneous distribution of points is apparent when plotting geographical distances against genetic ones (Figure S2), indicating that the genetic structure of Italy is better characterised by discontinuities than by clinal patterns.

These general spatial patterns were further explored by means of sPCA based on haplogroup frequencies. The analysis showed that the Italian genetic structure is characterised by two significant global components (positive eigenvalues) with similar variance values, being sPC1 characterized by a higher spatial autocorrelation (Moran's I) (Figure S3). These observations are further assessed by means of a significant Global test (observed value  = 0.08, p-value  = 0.015) and a non-significant Local test (observed value  = 0.06, p-value  = 0.677).

**Figure 1:**
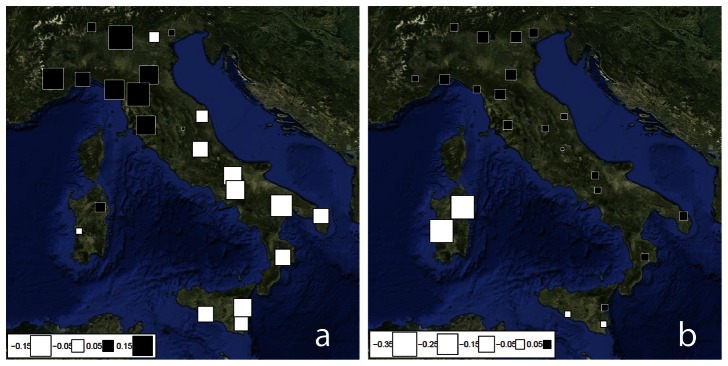
Spatial Principal Component Analysis (sPCA) based on frequencies of Y-chromosome haplogroups. The first two global components, sPC1 (a) and sPC2 (b), are depicted. Positive values are represented by black square; negative values are represented by white squares; the size of the square is proportional to the absolute value of sPC scores.

Geographical patterns of sPC1 and sPC2 are plotted in Figure 1. sPC1 identifies two main groups of populations separated by an almost longitudinal line (Figure 1a). The first group (black squares) is represented by populations from North-Western Italy, including most of the Padana plain and Tuscany. The second group (white squares) includes locations from South-Eastern Italy and the whole Adriatic coast, being represented also in North-Eastern Italy. Nonetheless, these two groups are not separated by a sharp discontinuity, but by some sort of gradient, as it is represented by a few samples from North-Eastern and Central Italy that show very low absolute values of sPC1 scores. However, sPC2 scores differentiate Sardinia from the rest of Italy (Figure 1b). Indeed, scores from these populations show the highest absolute values, while those from the other Italian locations (especially in the South) are much lower. In summary, sPC1 and sPC2 depict a three-partitioned structure of Italian population: 1) North-Western Italy (from now on NWI), 2) South-Eastern Italy (from now on SEI), and 3) Sardinia (from now on SAR).

When we tested the reliability of these three groups (NWI, SEI, SAR), by means of AMOVA based both on haplogroup frequencies and STR variability, the proportion of variation between groups (haplogroup frequencies: 3.71%; haplotypes: 4.48%; both p-values <0.001; Table S4) was 1.5 times higher than the variation explained when grouping according to the eight sampling macro-areas (2.62%, p-value <0.001, and 3.11%, p-value <0.001, respectively, Table S4). Interestingly, there is a partial congruence between sPCA-based groups and sampling macro-areas (Figure S1). In particular, SAR coincides with macro-area 8, while macro-areas 1, 3 and 4 are grouped in NWI and macro-areas 6 and 7 are grouped in SWI; macro-areas 2 and 5 are crossed by the sharp gradient that separate NWI from SEI.

To further test the reliability of the mentioned structure, for each of the considered populations we calculated DAPC-based posterior membership probabilities to the considered three groups. Results (Table S5) show that all the populations are characterised by high congruence (membership probability  = ∼9% or higher) to the given SPCA-group, the only exception being a single population from Central Italy (Foligno/PG), whose intermediate position between NWI and SEI has been already revealed by sPCA.

Interestingly, NWI revealed a high and significant degree of internal differentiation, while SEI is a fairly homogeneous group (Fst = 0.014, p-value <0.001 and Fst = 0.002, p-value >0.05, respectively; both estimates are based on haplogroup frequencies).

In order to quantify the contribution of each haplogroup to the genetic structure detected, the loadings values of the sPC1 and sPC2 were calculated and plotted in Figure S4. Lineages contributing more to the differentiation along the first sPC were R-U152*, G-P15 and, with lower loadings values, R-L2* and R-P312* (Figure S4a). On the contrary, sPC2 is influenced primarily and almost exclusively by the haplogroup I-M26 (Figure S4b).

#### Haplogroup DAPC analysis

**Figure 2:**
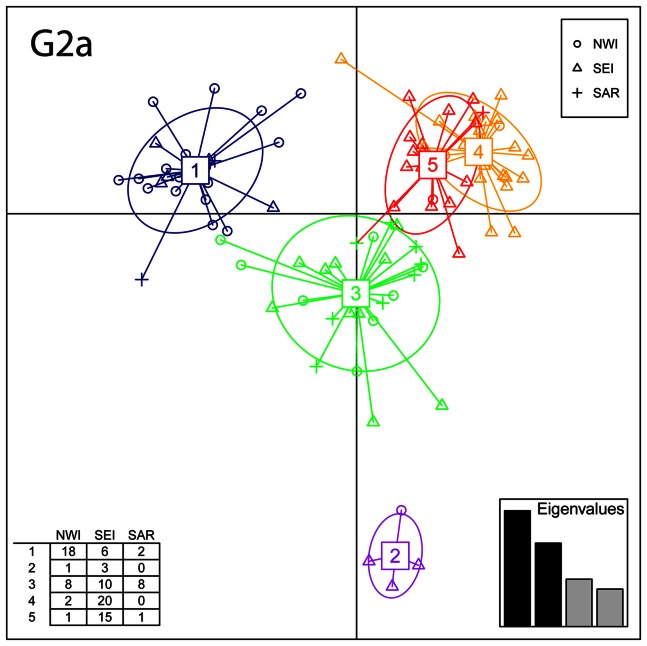
Discriminant Analysis of Principal Components (DAPC) for G2a-P15 haplotypes. Samples are grouped according to their affiliation at the sPCA-identified groups (NWI; SEI; SAR; symbols in the top right table). The table in the bottom left shows the number of haplotypes in each of the five G2a clusters and their geographical distribution in the three Italian areas. DAPC eigenvalues are depicted in the enclosed barplot.

DAPC was performed within the most frequent haplogroups (E-V13, G-P15, I-M26, J-M410*, R-P312*, R-U152*, R-L2*). Results (Table 1, Figure 2, Figure S5) show how the seven considered haplogroups disaggregate in 25 clusters, ranging from a minimum of two (I2a-M26) to a maximum of five (E-V13, G2a-P15). Considering a 70% threshold, 13 out of 25 are mostly frequent in one of the sPCA-identified areas (NWI: 7, SEI: 4, SAR: 2) (Table 1).

**Table 1:** Frequencies of Y-Chromosome DAPC cluster for each Italian sPCA-identified group.

HG	DAPC CLUSTER	N. HAPLOTYPES				N. INDIVIDUALS				MAX% (GROUP)
		NWI	SEI	SAR	TOT	NWI	SEI	SAR	TOT	
**E-V13**	1	8	10	1	19	8	10	1	19	53% (SEI)
	2	6	6	0	12	6	6	0	12	50% (NWI, SEI)
	**3**	3	11	1	15	3	**11**	1	15	**73% (SEI)**
	4	5	6	0	11	5	6	0	11	55% (SEI)
	5	6	6	0	12	6	6	0	12	50% (NWI, SEI)
**G2a-P15**	1	18	6	2	26	**20**	6	2	28	**71% (NWI)**
	2	1	3	0	4	1	3	0	4	75% (SEI)*
	3	8	10	8	26	8	10	8	26	38% (SEI)
	**4**	2	20	0	22	2	**20**	0	22	**91% (SEI)**
	**5**	1	15	1	17	1	**16**	1	18	**89% (SEI)**
**I2a-M26**	**1**	0	1	18	19	0	1	**19**	20	**95% (SAR)**
	**2**	2	1	12	15	2	1	**13**	16	**81% (SAR)**
**J2a-M410**	1	7	9	3	19	7	9	3	19	47% (SEI)
	2	8	18	2	28	8	19	2	29	66% (SEI)
	3	7	11	0	18	7	12	0	19	63% (SEI)
**R-P312**	**1**	11	4	1	16	**12**	4	1	17	**71% (NWI)**
	2	13	8	0	21	13	9	0	22	59% (NWI)
	3	6	5	0	11	6	5	0	11	55% (NWI)
**R-U152**	1	16	7	2	25	16	7	2	25	64% (NWI)
	**2**	21	1	0	22	**21**	1	0	22	**95% (NWI)**
	3	23	8	2	33	24	10	2	36	67% (NWI)
	**4**	16	4	2	22	**17**	5	2	24	**71% (NWI)**
**R-L2**	**1**	18	1	1	20	**18**	1	1	20	**90% (NWI)**
	**2**	18	6	1	25	**18**	6	1	25	**72% (NWI)**
	**3**	10	4	0	14	**10**	4	0	14	**71% (NWI)**

*Number of individuals <10

The absolute number of haplotypes and individuals are shown for each DAPC-cluster, and the maximum frequency for each cluster is expressed in percentage (max%). NWI: North-Western Italy; SEI: Southern and Eastern Italy; SAR: Sardinia.

It is noteworthy the structure shown by haplogroup G2a-P15 (Figure 2), which includes clusters with very different spatial distribution: cluster 1 is mostly frequent in NWI, while clusters 4 and 5 – partially overlapping in the DAPC plot – are found in SEI. For comparison purposes, we calculated a Median Joining Network (Figure S6) based on the same haplotypes. While results from both methods are largely overlapping, DAPC offers some advantages compared to the network, namely 1) it outputs clear-cut clusters (while in Network the definition of clusters is in some way arbitrary), 2) it gives probability memberships for each individual. Networks for other haplogroups are not shown.

DAPC comparisons with additional samples (Table S6, Figure S7) suggest differential affinities for some of the considered haplogroups and clusters of haplotypes. Most notably, G2a-P15 haplotypes from NWI cluster mainly with German ones, while haplotypes from SEI seem to indicate wider relationships, going from Iberia to the Balkans and the Caucasus. On the contrary, I2-M26 samples from Sardinia (SAR) cluster in a separate group than Iberians, suggesting a geographical neat separation between continental and Sardinian I2-M26 lineages.

#### Date estimates for paternal variation

BATWING was used to estimate the age of split between the Italian regions identified by the first sPCA (NWI and SEI, excluding SAR). BATWING modelled population growth starting at 12,890 YBP (95% CI: 3,700–83,070), with a rate of 0.00429 (95% CI: 0.00254–0.01219) per year. Our results suggest that the split happened around 5,490 YBP (95% CI: 1,620–26,830). Since BATWING does not consider migration, admixture between NWI and SEI would likely bias the split time estimate towards more recent dates.

Concerning Y-chromosome lineages, STR variation within the 13 clusters mentioned above suggests that most of them date back to relatively recent times (Table 2). In fact, the ages of the considered clusters (with a peak in one of the considered sPCA groups) fall roughly within the interval from the time of split estimated with BATWING between NWI and SEI and the present. This is consistent with the fact that group-specific clusters of haplotypes (NWI, SEI) are very likely to have emerged after the split within the Italian ‘ancestral’ population or later. No different patterns of timing are detected between both regions. The time estimates were similar for whole haplogroups with the notable exception of G2-P15, which showed older ages. These results suggest that most of the Y-chromosomal diversity present in modern day Italians was originated from few common ancestors living during late Neolithic times and the Early Metal Ages. However, if we would take into account evolutionary rates, we would observe results three times higher than those above mentioned, meaning that most dates would shift to late Paleolithic.

**Table 2:** Age estimates (in YBP) of STR and HVS variation for the most common haplogroups in the Italian data set.

Y Chromosome Haplogroups	SD	SE	Age estimate	SE
**E-V13**	146.46	51.78	3662	1295
Cluster3 (SEI 70.3%)	139.52	49.33	3488	1233
**G-P15**	600.79	212.41	15020	5310
Cluster1 (NWI 71.4%)	144.31	51.02	3608	1276
Cluster3	505.72	178.80	12643	4470
Cluster4 (SEI 90.9%)	111.40	39.39	2785	985
Cluster5 (SEI 88.9%)	240.62	85.07	6016	2127
**I-M26**	206.11	72.87	5153	1822
Cluster 1 (SAR 95.0%)	48.26	17.06	1207	427
Cluster 2 (SAR 81.3%)	227.81	80.54	5695	2014
**R-U152**	137.29	48.54	3432	1214
Cluster2 (NWI 95.5%)	199.16	70.41	4979	1760
Cluster4 (NWI 70.8%)	184.29	65.16	4607	1629
**R-L2**	129.67	45.85	3242	1146
Cluster1 (NWI 90.0%)	250.32	88.50	6258	2213
Cluster2 (NWI 72.0%)	185.52	65.59	4638	1640
Cluster3 (NWI 71.4%)	148.55	52.52	3714	1313
**R-P312**	302.55	106.97	7564	2674
Cluster1 (NWI 70.6%)	130.05	45.98	3251	1149

mtDNA Haplogroups	Rho	SE	Age estimate	SE
H*	1.79	0.16	16229	2889
**H1_whole (including all H1 derivates)**	1.72	0.15	15604	2588
H1*	1.43	0.14	12983	2549
**H3**	1.71	0.28	15452	4954
**H5**	1.62	0.23	14689	4015
**HV**	3.49	0.33	31574	5872
Cluster 2 (NWI 75%)	2.00	0.42	18116	7476
Cluster 3 (SEI 85%)	2.33	0.39	21135	7002
**U5a**	3.13	0.35	28306	6128
**K1a**	1.84	0.25	16686	4383
Cluster 2 (NWI 71%)	1.33	0.28	12077	4929
**J1c**	2.15	0.27	19448	4757
				

Standard deviation (SD) estimator (Sengupta et al. 2006) and ñ statistic calculator (Soares et al. 2009) were used for Y-chromosome and mtDNA haplogroups respectively. Ages were estimated for the entire haplogroups as well as for each DAPC cluster with at least 10 individuals and frequencies >70% in NWI, SEI, or SAR (excepted for G-P15, cluster 2, see Methods).

### Mitochondrial DNA lineages in Italy

#### Haplogroup frequencies

The maternal genetic ancestry of Italian populations was explored by characterizing coding region SNPs and control region sequences from 865 individuals, which yielded to 79 distinct mtDNA haplogroups (including sublineages). Haplogroup frequencies and within-population diversity parameters are shown in Table S7 and Table S3 respectively.

The haplogroup distribution in Italy reflects the typical pattern of mtDNA variability of Western Europe. As described for other European and Italian populations [2], [6], [14], [15], [57] most of the sequences belong to the super-haplogroup H, which includes 44.4% of the Italian mtDNA lineages. In particular, H1 turned out to represent a large proportion of H samples, encompassing the 13.8% of the total variability (10.4% excluding sub-lineages). Compared to H1, sub-haplogroups H3 and H5 represent much smaller fractions of H composition, reaching however noteworthy frequencies (3.9% and 4.3% respectively). Most of the remaining samples belong to haplogroups frequently found in western Eurasia, including U5, K1, J1, J2, T1, T2, and HV. Among the U5 lineages, U5a is the most frequent (3.70%). Haplogroups K1a, HV and J1c take into account respectively the 4.39%, 4.05% and the 3.93% of the total mtDNA variability. The remaining lineages reach frequencies that do not exceed a 3.5% threshold.

#### Maternal population structure

In contrast to paternal lineages, correlation between geographical and genetic distances was non-significant (Mantel Test: observed value  = 0.011, p-value  = 0.45). These results point to a strong homogeneity within the Italian Peninsula for the mtDNA gene pool composition. In order to extract further insights into the distribution of mtDNA lineages, a sPCA was performed using haplogroup frequencies. The highest absolute eigenvalues (Figure S8) correspond to the first two positive components (global structure). According to the Global test of significance, the geographical distribution of the genetic variability observed with sPCA was found to be marginally significant (observed value  = 0.061, p-value  = 0.046).

**Figure 3:**
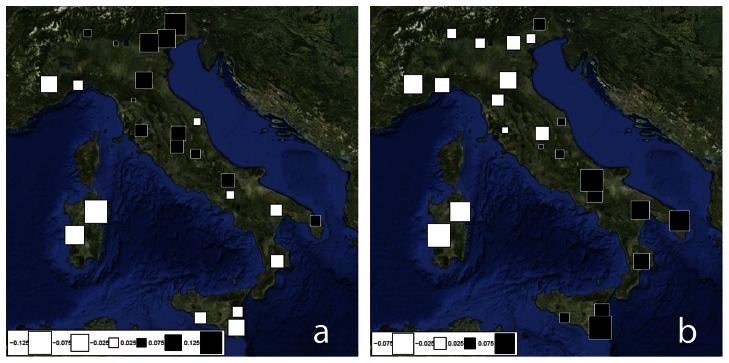
Spatial Principal Component Analysis (sPCA) based on frequencies of mtDNA haplogroups. The first two global components sPC1 (a) and sPC2 (b) are depicted. Positive values are represented by black squares; negative values are represented by white squares; the size of the square is proportional to the absolute value of sPC scores.

Scores of the sPC1 and sPC2 are plotted in Figure 3. Both sPC1 and sPC2 highlight the extreme position of Sardinia (large white squares). In addition, sPC1 identifies a North-East centred group that spreads southwards along the Apennines (including most of populations from central Italy), while sPC2 highlights the same East-West pattern observed for Y-chromosome. Loadings of sPC1 and sPC2 (Figure S9) identify lineages H1 and H3 respectively as the haplogroups affecting more the spatial genetic differentiation of Italian populations.

#### Haplogroup DAPC analysis

DAPC was performed within the eight most frequent haplogroups (H*, H1, H3, H5, HV, J1c, K1a, U5a). They disaggregate in 24 haplotype clusters (Table S8, Figure S10), ranging from a minimum of two (K1a) to a maximum of four (U5a). Most of them are widespread in the whole of Italy, in fact, if we consider a 70% threshold, only nine clusters show traces of geography-related distributions (but six of them are composed by less than 10 individuals). Haplogroup HV is the most important exception, including two clusters located in NWI and SEI, respectively. It is noteworthy a cluster from haplogroup H3 that is almost exclusive of SAR.

Comparisons with other European samples (Table S9, Figure S11) confirm that great part of Italian mtDNA haplotypes share a wide range of affinities spanning from Iberia to Eastern Europe, but haplotypes from H1 and H3 appear to be related mostly with Western and Central Europe.

#### Date estimates for maternal variation

TMRCA estimates for the most frequent haplogroups (Table 2) could be classified in two groups: “old” haplogroups, predating the Last Glacial Maximum, LGM (∼31,600 YBP for HV, ∼28,300 YBP for U5a and ∼19,500 YBP for J1c), and haplogroups dating after the LGM (∼16,200 YBP for H*, ∼15,600 YBP for H1, ∼15,500 YBP for H3, ∼14,700 YBP for H5, ∼16,700 YBP for K1a). Estimates for H1 and H3 haplogroups are slightly older than estimates in Western Eurasia for the same haplogroups [2], [4], [5], [55]. These results are in agreement with what has been shown for the Basque region in Iberia [27] and may be related to the length of the mitochondrial region used.

Additionally, we calculated TMRCA for the two DAPC clusters within HV haplogroup (2 and 3), given that they show a clear spatial polarity within continental Italy and Sicily. Their ages fall between the time estimate for the whole haplogroup (∼31,600 YBP) and the LGM, suggesting that their differentiation happened during this time frame (Table 2).

## Discussion

Previous reconstructions of the genetic structure of Italy agreed on two points: the peculiarity of the population of Sardinia – due to a distinct background and a high degree of isolation [58], [59] – and the clinal pattern of variation in the Italian Peninsula, which has been explained by differential migration patterns [17], [18] although some genetic discontinuity due to local drift and founder effects have been described [16], [19], [20]. This study represents a significant upgrade on the knowledge of the genetic structure of Italy for the following reasons: the wide sampling coverage (coupled to a detailed sampling strategy), the high number of typed markers and the innovative methodological approach. Our results show that the Y-chromosomal genetic diversity of Italy is not clinal but structured in three geographical areas: North-Western Italy (NWI), South-Eastern Italy (SEI) and Sardinia (SAR). The outlier position of SAR described in previous studies [21], [58]–[61] is mainly due to the high frequency of I-M26 haplogroup, that in turn is almost completely absent in continental Italy. In addition, it is noteworthy the scanty haplotype affinities with other European I-M26 lineages as DAPC results seem to indicate (Figure S7, Table S6). However, the structure observed for paternal lineages in continental Italy and Sicily was not characterised by North-South gradients as previously described: our results show a NWI-SEI clustering (Figure 1a), suggesting a shared genetic background between Southern Italy and the Adriatic coast from one side, and between Northern Italy and Tuscany from the other side. Actually, the most accurate description of the discontinuity between NWI and SEI is that of a “belt”, that is a restricted portion of territory in which haplogroup frequencies tend to change more rapidly than in the rest of the Italian peninsula. This model was suggested by the presence of a few populations from North-Eastern and Central Italy (Treviso, Foligno/PG) that reveal an intermediate position between the two main groups.

The discontinuous Y-chromosomal structure of continental Italy is also confirmed by the distribution of DAPC haplotype clusters identified for the most frequent haplogroups (Table 1). Haplogroup G2a provides the most compelling case, being widespread in the whole region, but revealing different clusters in NWI and SEI (Figure 2). This is in agreement with a recent G haplogroup survey that revealed the presence of different G2a sub-clades in Italy [62]. Nevertheless, we are not identifying the whole Italian population history with a single haplogroup. In fact, comparisons with other populations taking into account the whole haplogroup spectrum suggest differential patterns of haplotype similarity, implying different genetic histories for the identified sPCA-groups. In particular, NWI is mostly related with Western and Central Europe, while SEI seems to indicate more affinities with the Balkans. In addition, NWI and SEI are characterised by different distributions of genetic variance, the latter showing higher intra-population and lower (not significant) inter-population variability, while the opposite is true for NWI, where significant variation between populations was detected. On the whole, these patterns may be explained by a higher degree of population mobility in SEI, while in NWI local drift effects may have had a greater impact.

In contrast to the results obtained for Y-chromosome, the mtDNA diversity in Italy is characterised by a high degree of homogeneity: the only exception (a marginally significant sPCA global test based on haplogroup frequencies) is due to significant differentiation found in the Sardinian samples compared to continental Italy and Sicily (AMOVA difference between groups  = 1.02%, p<0.05, Table S4). These results (in agreement with Y chromosome) suggest at least partially different demographic histories for SEI-NWI populations on one hand and SAR on the other hand, the latter being less affected to the gene flow of different migrations occurred in the Italian Peninsula and Sicily. Traces of such processes are visible in sPCA results (Figure 3) and in particular in sPC2, reflecting the same NWI-SEI pattern shown by Y-chromosomal sPC1. Anyway, such differentiation was not significant in the case of mtDNA (AMOVA difference between groups  = 0.10%, p = 0.08). Analogously, DAPC clusters of mtDNA haplotypes do not show any geographic structure even when compared with other European samples, with clusters of similar haplotypes spanning from Iberia to the Balkans. However, not only uniparental differences in the genetic structure but also in time estimates are shown in the present dataset: our age estimates for the Y-chromosome and the mtDNA haplogroups (as well as the corresponding clusters of haplotypes) highlight significantly different time periods (Table 2), which could reflect multi-layered histories in Italy. Age estimates for mtDNA haplogroups - even if past demographic events affecting error rates cannot be excluded - point almost unanimously to pre-Neolithic times, ranging approximately from ∼13,000 (H1*) to ∼31,600 (HV) YBP. Although such estimates might reflect the haplogroups pre-existent diversity previous to their establishment in Italy (which could be the case of HV, that includes two DAPC clusters with different geographical distributions and whose ages largely post-date that of the whole haplogroup; Table 2), this does not seem to hold for most of the mtDNA haplogroups analysed. Indeed, most of our mtDNA time estimates are consistent with the hypothesis of the existence of a Glacial Refugium in the Italian Peninsula and its probable role in subsequent post-glacial expansions.

Actually, the role of Italy as a Southern European Glacial Refugium – together with the Iberian and Balkan peninsulas – is demonstrated for a high number of animal and plant species [63]–[69]. The presence of numerous Epigravettian sites suggests strongly that Italy could have acted as such also for humans [70]. Nevertheless, molecular evidences going in the same direction are still scarce, the only exception being mitochondrial haplogroup U5b3 [8], [9] whose frequency in Italy is relatively low (U5b lineages account for 1.73% in our data). Our results suggest that most of Italian mitochondrial diversity originated during and immediately after LGM. In particular, estimates for H1 and H3 are even older in Italy than in the Franco-Cantabrian area [27] where these clades have been postulated to originate [4]. Furthermore, DAPC comparisons with a wide set of European haplotypes (Table S9) show that Italy, in most cases, is characterised by the highest number of different haplotypes. On the whole, these observations not only are in agreement with the existence of a human Glacial Refugium in Italy, but also suggest that its relevance has been until now largely underrated.

The use of STR variation for dating Y-chromosome lineages or population splits, is a controversial issue, due to the effect that both mutation rates and STR choice has on the temporal scale of age estimates. Following the most recent studies our estimates are based on those STRs that show the highest duration of linearity [49] and by using locus-specific mutation rates (Ballantyne et al. 2010). This is one of the reasons that led us to exclude ‘evolutionary’ mutation rates (see Methods for details). In addition, we removed ‘outlier’ haplotypes (see Methods S1), since their presence could inflate significantly the ages of haplogroups and DAPC clusters. However, these results have to be taken with great caution, keeping in mind that ‘evolutionary’ rates (applied to the same data) would yield time estimates around three times greater. Nonetheless, we observe that two independent methods applied to our data – BATWING and SD-based estimates – yield consistent results. In fact, in contrast to mtDNA age estimates, almost all Y-chromosome estimates fall between late Neolithic and the Bronze Age. This finding supports the hypothesis that group-specific clusters of haplotypes did originate after the split between NWI and SEI (dated with BATWING), even if the confidence interval for BATWING estimate is not tight enough to exclude alternative hypotheses. Interestingly, the NWI and SEI structure detected (Figure 1, Table S4) might be traced back around 5,500 YBP indicating relevant demographic events within continental Italy in this period. Anyway, this value has to be considered as a lower bound, given that the model used does not account for migration that would bias the split time towards recent dates. In fact, given a specific level of populations differentiation, the separation time estimated between these populations has necessarily to be higher (i.e. more ancient) as migration is considered.

According to the most recent syntheses, the Neolithic revolution diffused in Italy following two independent routes along the Adriatic (Eastern) and the Tyrrhenian (Western) coasts. Furthermore, archaeological sites from NWI are characterized by a deeper continuity with earlier Mesolithic cultures and a higher degree of local variability than SEI, while this last area, besides being culturally more homogeneous, shows clear links with the Southern Balkans [10]. Our Y-chromosome results – showing discontinuity between NWI and SEI, higher inter-population variability in NWI, higher homogeneity in SEI coupled with relevant contributes from the Balkans – are quite consistent with this model. Thus, we can hypothesize that the NWI-SEI structure detected with paternal lineages could have its origins after these different Neolithic processes. Indeed, comparisons with other European and Near-Eastern populations (Table S6) suggest a stronger affinity between NWI with Iberia and Central Europe, while SEI is more related to the Balkans and Anatolia. The emergence of population structures during the Neolithic has been recently shown in two different studies using Y-chromosome markers, in Near East [71] and in Western Europe [27]. Our results confirm these findings and emphasize the role of demographic expansions and cultural advances related to the Neolithic revolution in shaping human genetic diversity, at least for male lineages. Nonetheless, such pattern might have been further influenced and/or re-shaped also by more recent events.

For instance, the dates of several DAPC clusters fall within the range of the Metal Ages (Table 2). During this long period (third and second millennia BC) Italy underwent important technological and social transformations finally leading to the ethnogenesis of the most important proto-historic Italic peoples. On the whole, our results indicate that these transformations, far from being exclusively cultural phenomena, actually involved relevant population events.

It is worth noting the older age estimate obtained for Y-haplogroup G2-P15 (15,020 YBP) that, coupled with its high frequency (11.09%), makes it the most probable candidate for a continuity with Italian Mesolithic populations (although a Neolithic origin for G2-P15 is discussed, [22], [23]). The most frequent G2-P15 cluster (12,643 YBP, Table 2), besides being evenly diffused in NWI and SEI, it encompasses almost all Sardinian G2-P15 individuals (Figure 2, Table 1). These facts, together with the higher degree of isolation of Sardinia to Neolithic and Post-Neolithic migration processes, support the antiquity of this haplogroup in Italy. Despite obtaining similar time estimates for G2a in Italy (12,899 YBP), Rootsi et al. (2012) [62] explain the diffusion of its main sub-lineages in this country solely as a consequence of Neolithic and Post-Neolithic events.

## Conclusions

This study depicts the most complete picture of Italian genetic variability from the point of view of uniparental markers to date. Our analyses revealed that the Y-chromosomal genetic structure of Italy is characterised by discontinuities. Such a structure is defined by three different and well-defined groups of populations: the Sardinia island (SAR), North-Western Italy (NWI) and South-Eastern Italy (SEI). Furthermore, we observed that NWI and SEI are not separated according to latitude but following a longitudinal line. Such discontinuity may date at the Neolithic revolution in Italy, which was characterised by (at least) two independent diffusion processes involving the Western and Eastern coasts, respectively. Mitochondrial DNA, despite showing some correspondence with Y-chromosome results, depicts a substantially homogeneous genetic landscape for the Italian peninsula. Significantly different ages were estimated for mtDNA and Y-chromosome systems. mtDNA variability dates back to Paleolithic and supports the existence of an Italian human Refugium during the last glacial maximum whereas Y-chromosome points to the importance that the demographic events happened during the Neolithic and the Metal Ages had in the male Italian patterns of diversity and distribution.

## Supporting Information

Figure S1 **Map showing the geographical location of populations sampled in the present study.** Colors indicate the eight clusters of homogeneous Italian provinces (sampling macro-areas) identified after a preliminary surname-based analysis [24]. The set of provinces (sampling points) and the number of samples successfully typed for Y-chromosome and mtDNA markers are detailed for each sampling macro-area (table on the left).
(TIF)

Figure S2 **Plot of geographical distances against genetic distances (based on frequencies of Y-chromosome haplogroups).** A 2-dimensional kernel density estimation layer (Venables and Ripley 2002) was added to the plot. The analysis was performed including (a) and excluding (b) the Sardinian samples.
(TIF)

Figure S3 **Eigenvalues of Y-chromosome-based sPCA analysis (A) with their decomposition in spatial and variance components (B).** Eigenvalues are obtained maximizing the product of variance and spatial autocorrelation (Moran's I index). They are both positive and negative depending from Moran's I positive or negative values. Large positive components correspond to global structures (cline-like structures); large negative components correspond to local structures (marked genetic differentiation among neighbours).
(TIF)

Figure S4 **Loadings of the most informative components (a: sPC1, b: sPC2).** These values identify Y-chromosome haplogroups that mostly affect the genetic structure of Italian populations.
(TIF)

Figure S5 DAPC analysis of STRs variation for the most frequent Italian Y-chromosome haplogroups (E-V13, I-M26, J-M410, R-P312*, R-U152*, R-L2). Samples are grouped according to their affiliation to sPCA-identified areas (NWI, SEI, SAR; symbols in the top right legend of each plot). For each plot, the number of different haplotypes per cluster and their geographic distribution in the above areas are shown in the enclosed table. The DAPC eigenvalues are depicted in the enclosed barplot. Haplogroup I-M26, including two clusters only, is represented by a single discriminant function (no eigenvalues barplot).
(TIF)

Figure S6 **Median joining network for Italian G2a-P15 haplotypes.** Individuals have been assigned and colored according to the correspondent DAPC-based clusters (Figure 2).
(TIF)

Figure S7 **DAPC analysis of STRs variation for the most frequent Y-chromosome haplogroups.** Results are based on Italian data and additional comparison samples (NWI; SEI; SAR; IBE: Iberian Peninsula; BAL: Balkan Peninsula; GER: Central-Europe (Germany); CAU: Caucasus; WAN: Western Anatolia; symbols in the legend of each plot). For each plot, the number of different haplotypes per cluster and their geographical distribution are shown in the enclosed table. The DAPC eigenvalues are depicted in the enclosed barplot.
(TIF)

Figure S8 **Eigenvalues of mtDNA-based sPCA analysis (A) with their decomposition in spatial and variance components (B).** Eigenvalues are obtained maximizing the product of variance and spatial autocorrelation (Moran's I index), and are both positive and negative, depending from Moran's I positive or negative values. Large positive components correspond to global structures; large negative components correspond to local structures (marked genetic differentiation among neighbours).
(TIF)

Figure S9 **Loadings of the most informative components (a: sPC1, b: sPC2).** These values identify mtDNA haplogroups that mostly influence the genetic structure of Italian populations.
(TIF)

Figure S10 DAPC analysis of HVS variation for the most frequent mtDNA haplogroups (H*, H1, H3, H5, HV, J1c, K1a, U5a) in the Italian data set. Results have been grouped geographically using the same categories as for Y-Chromosome (NWI; SEI; SAR); “0” codes were attributed to those populations for which Y-chromosome information was not available and whose geographical position lies along the boundary between NWI and SEI (Aviano, Terni). For each plot, the number of different haplotypes per cluster and their geographical distribution are shown in the enclosed table. The DAPC eigenvalues are depicted in the enclosed barplot. Haplogroup K1a, including two clusters only, is represented by a single discriminant function (no eigenvalues barplot).
(TIF)

Figure S11 **DAPC analysis of HVS variation for the most frequent mtDNAhaplogroups.** Results are based on Italian data and comparison European populations (ITA: Continental Italy; SAR: Sardinia; BASQ: Iberian Peninsula (Basques); AUST: Central Europe (Austria); MAC: Macedonians; ROM: Romanians; BALK: Balkan Peninsula; symbols in the legend of each plot). For each plot, the number of different haplotypes per cluster and their geographical distribution are shown in the enclosed table. The DAPC eigenvalues are depicted in the enclosed barplot.
(TIF)

Table S1 Frequencies of Y-chromosome haplogroups. Absolute values are reported for the whole Italian data set, while the frequencies within the eight sampling areas (from I to VIII) are expressed in percentage (%).
(XLS)

Table S2 Y-Chromosome STRs haplotypes in the 884 Italian samples of the present study.
(XLS)

Table S3 Diversity indices computed for the different Italian sampling points. Standard diversity parameters were calculated for both Y-chromosome and mtDNA based on haplotype/sequence data and haplogroup frequencies.
(XLS)

Table S4 Analyses of the molecular variance (AMOVA). Apportionment of the variance in %. Samples were grouped according to the geographic clusters (eight macro-areas) and to the sPCA results.
(XLS)

Table S5 DAPC membership probabilities to the SPCA-identified groups.
(XLS)

Table S6 Frequencies of Y-Chromosome DAPC clusters based on Italian data and comparison to other populations. The absolute number of haplotypes and individuals are shown for each population (NWI: sPCA North-Western Italy; SEI: sPCA Southern and Eastern Italy; SAR: Sardinia; IBE: Iberian Peninsula; BAL: Balkan Peninsula; GER: Central-Europe (Germany); CAU: Caucasus; WAN: Western Anatolia).
(XLS)

Table S7 Frequencies of mtDNA haplogroups. Absolute values are reported for the whole Italian data set, while the frequencies within the eight sampling areas (from I to VIII) are expressed in percentage (%).
(XLS)

Table S8 Frequencies of mtDNA DAPC clusters in Italy. Values were calculated both grouping according to the geographical clusters identified with Y-Chromosome sPCA (NWI: Y-sPCA North-Western Italy; SEI: Y-sPCA Southern and Eastern Italy; SAR: Sardinia) as well as considering the continental Italy (including Sicily) altogether (ITA). The absolute number of haplotypes and individuals are shown for each DAPC-cluster, and the maximum frequency for each cluster is expressed in percentage (max%).
(XLS)

Table S9 Frequencies of mtDNA DAPC clusters based on Italian data and comparison to other populations. The absolute number of haplotypes and individuals are shown for each population (ITA: Continental Italy and Sicily; SAR: Sardinia; BASQ: Iberia Peninsula (Basques); AUST: Central Europe (Austria); MAC: Macedonians; ROM: Romanians; BALK: Balkan Peninsula).
(XLS)

Methods S1 Spatial Principal Component Analysis (sPCA). Discriminant Analysis of Principal Components. Batwing analysis. “Jackknife-like” procedure for outliers identification.
(DOC)
