# Supplementary figures and images for: Uniparental Markers in Italy Reveal a Sex-Biased Genetic Structure and Different Historical Strata
Source: PLoS One. 2013 May 29;8(5):e65441. doi: 10.1371/journal.pone.0065441 (PMC3666984; doi:10.1371/journal.pone.0065441)

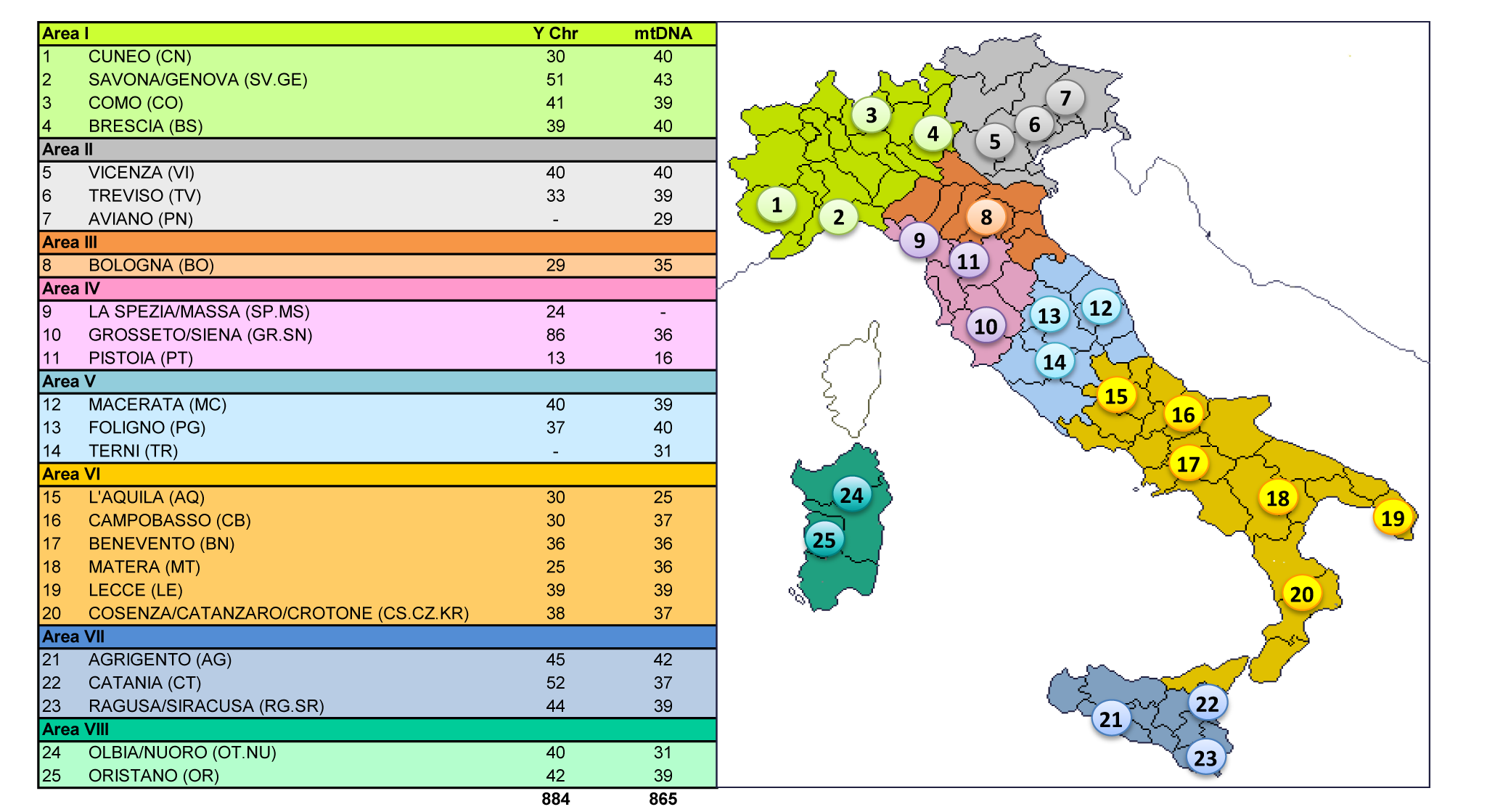

Supplement: Figure S1 — Map showing the geographical location of populations sampled in the present study. Colors indicate the eight clusters of homogeneous Italian provinces (sampling macro-areas) identified after a preliminary surname-based analysis [24]. The set of provinces (sampling points) and the number of samples successfully typed for Y-chromosome and mtDNA markers are detailed for each sampling macro-area (table on the left). (TIF) [file pone.0065441.s001.tif]

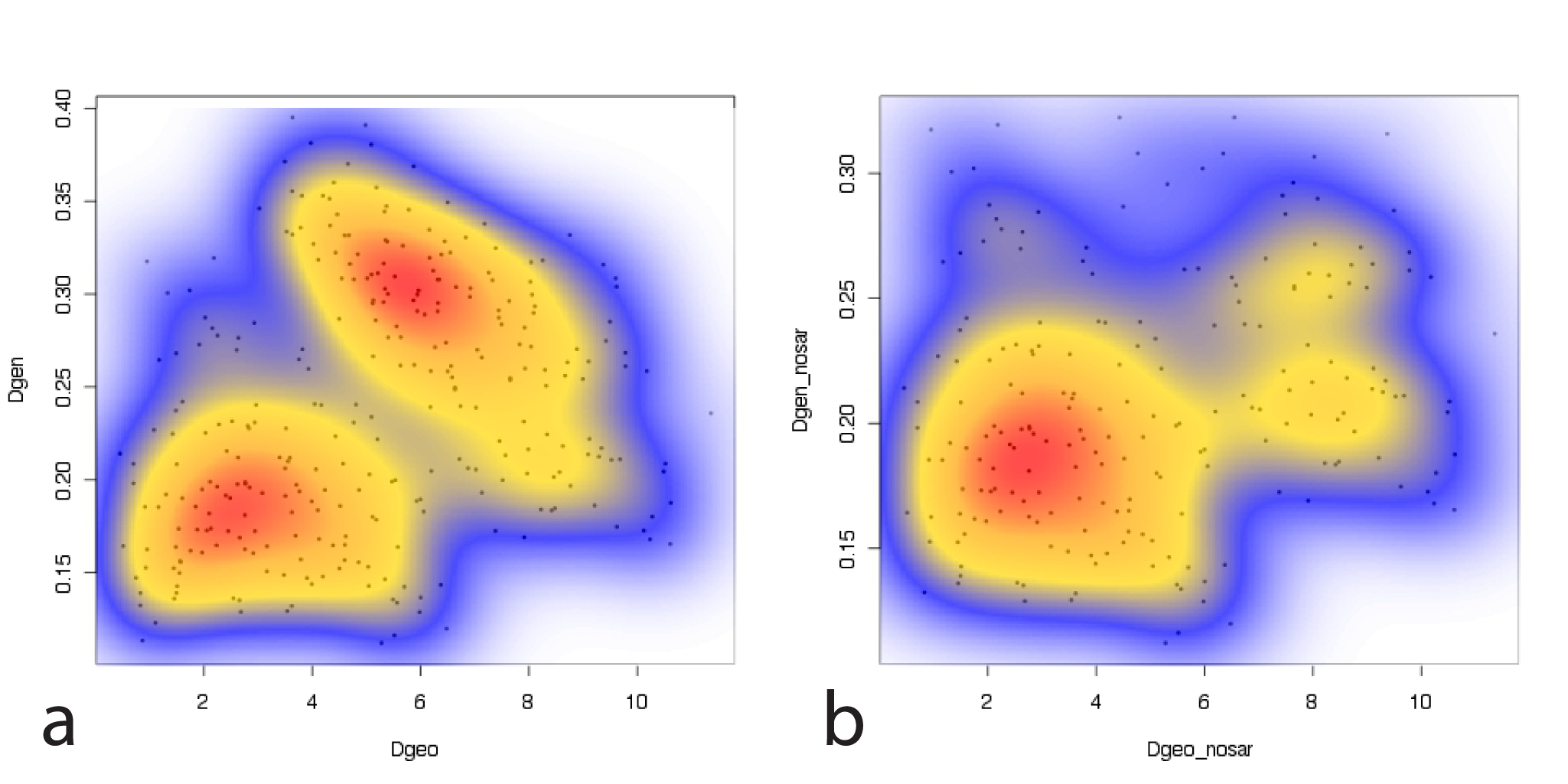

Supplement: Figure S2 — Plot of geographical distances against genetic distances (based on frequencies of Y-chromosome haplogroups). A 2-dimensional kernel density estimation layer (Venables and Ripley 2002) was added to the plot. The analysis was performed including (a) and excluding (b) the Sardinian samples. (TIF) [file pone.0065441.s002.tif]

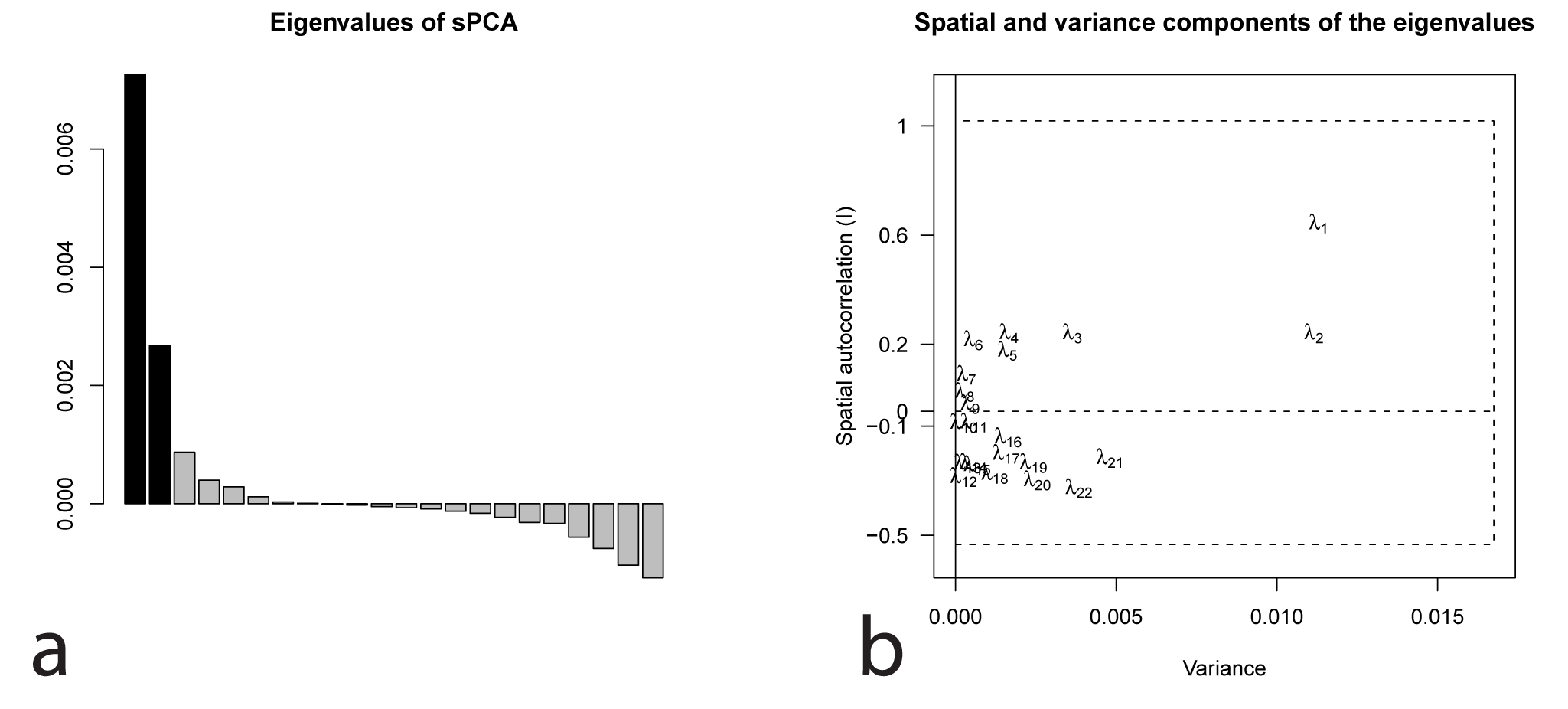

Supplement: Figure S3 — Eigenvalues of Y-chromosome-based sPCA analysis (A) with their decomposition in spatial and variance components (B). Eigenvalues are obtained maximizing the product of variance and spatial autocorrelation (Moran's I index). They are both positive and negative depending from Moran's I positive or negative values. Large positive components correspond to global structures (cline-like structures); large negative components correspond to local structures (marked genetic differentiation among neighbours). (TIF) [file pone.0065441.s003.tif]

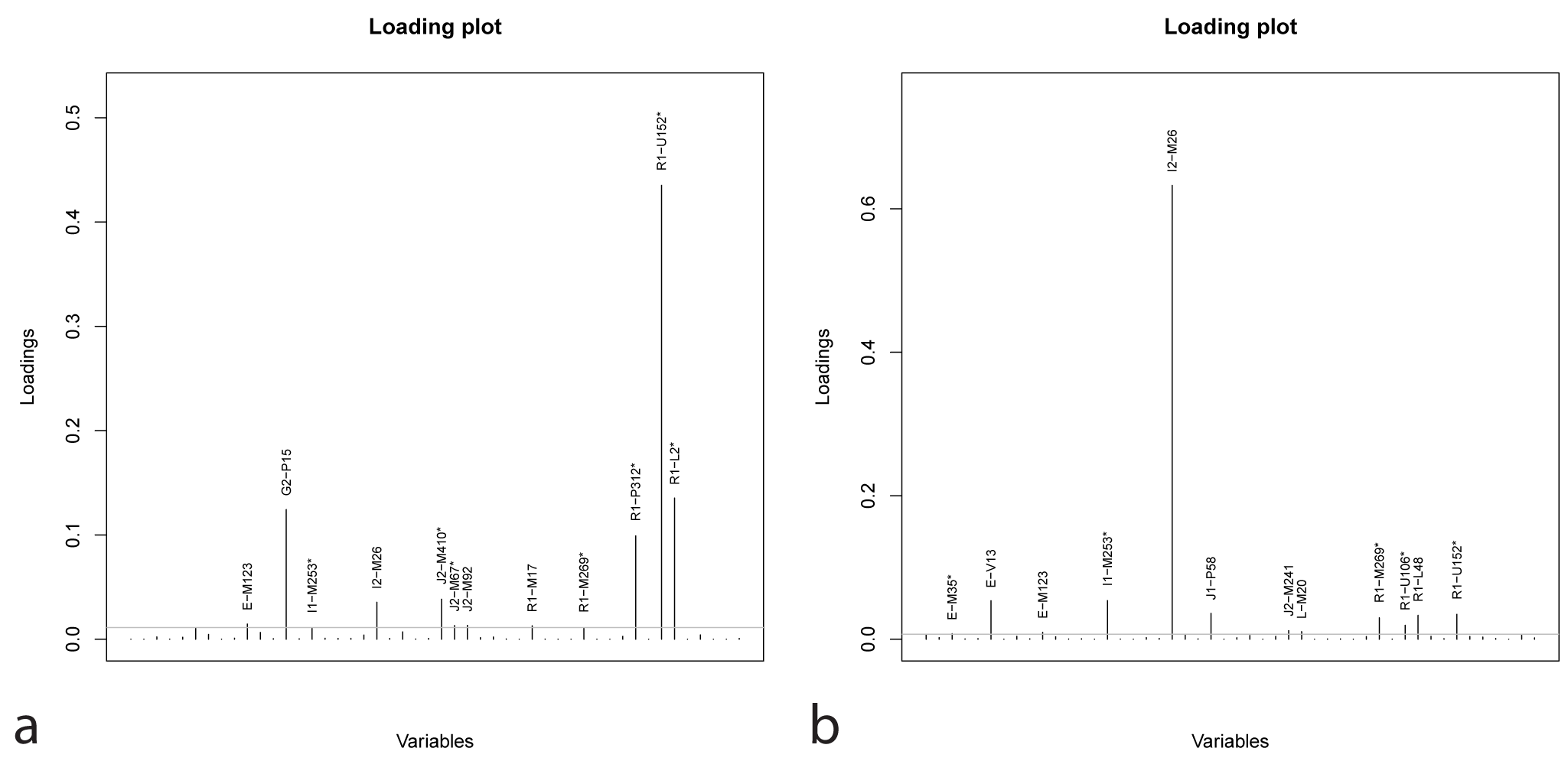

Supplement: Figure S4 — Loadings of the most informative components (a: sPC1, b: sPC2). These values identify Y-chromosome haplogroups that mostly affect the genetic structure of Italian populations. (TIF) [file pone.0065441.s004.tif]

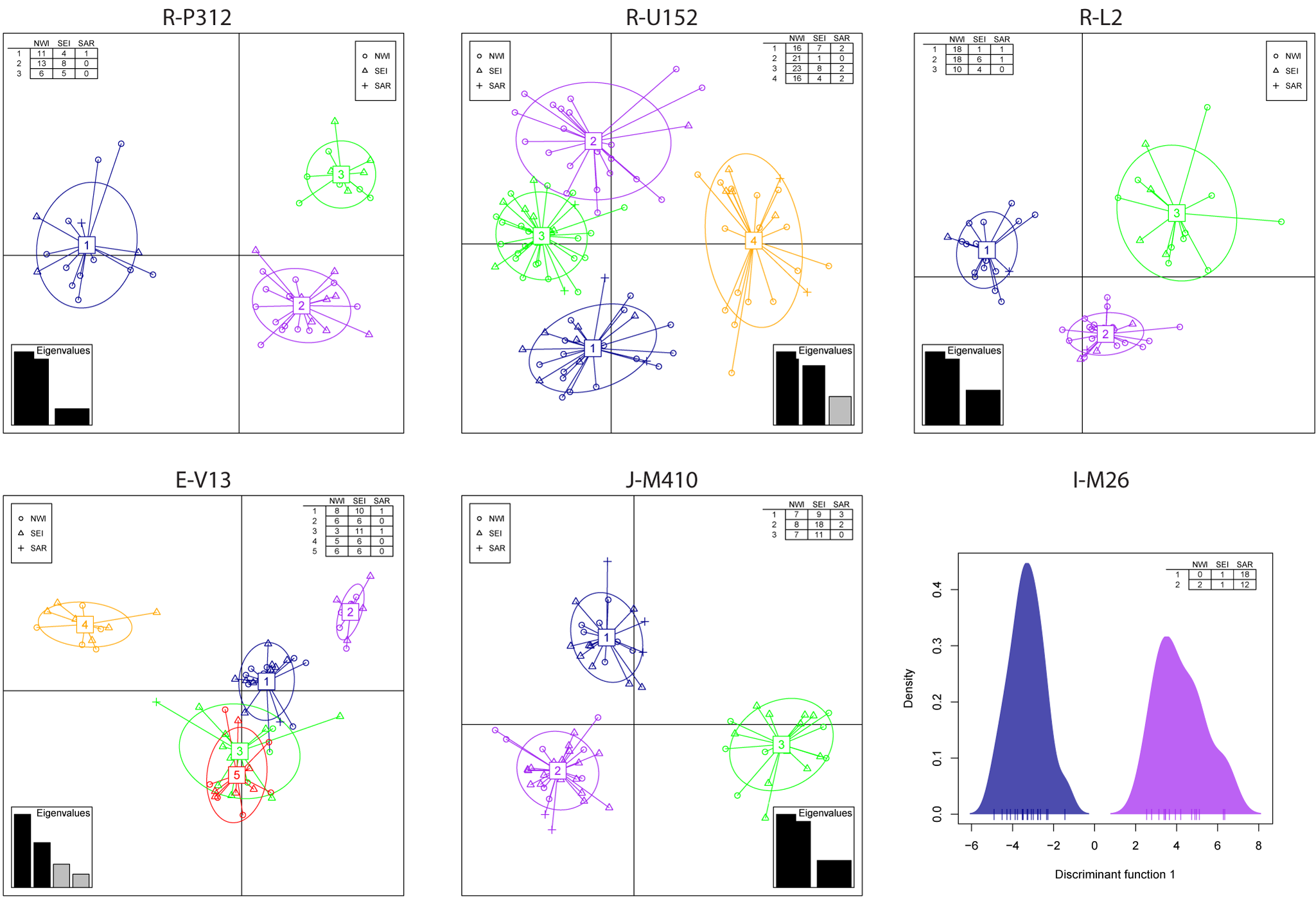

Supplement: Figure S5 — DAPC analysis of STRs variation for the most frequent Italian Y-chromosome haplogroups (E-V13, I-M26, J-M410, R-P312*, R-U152*, R-L2). Samples are grouped according to their affiliation to sPCA-identified areas (NWI, SEI, SAR; symbols in the top right legend of each plot). For each plot, the number of different haplotypes per cluster and their geographic distribution in the above areas are shown in the enclosed table. The DAPC eigenvalues are depicted in the enclosed barplot. Haplogroup I-M26, including two clusters only, is represented by a single discriminant function (no eigenvalues barplot). (TIF) [file pone.0065441.s005.tif]

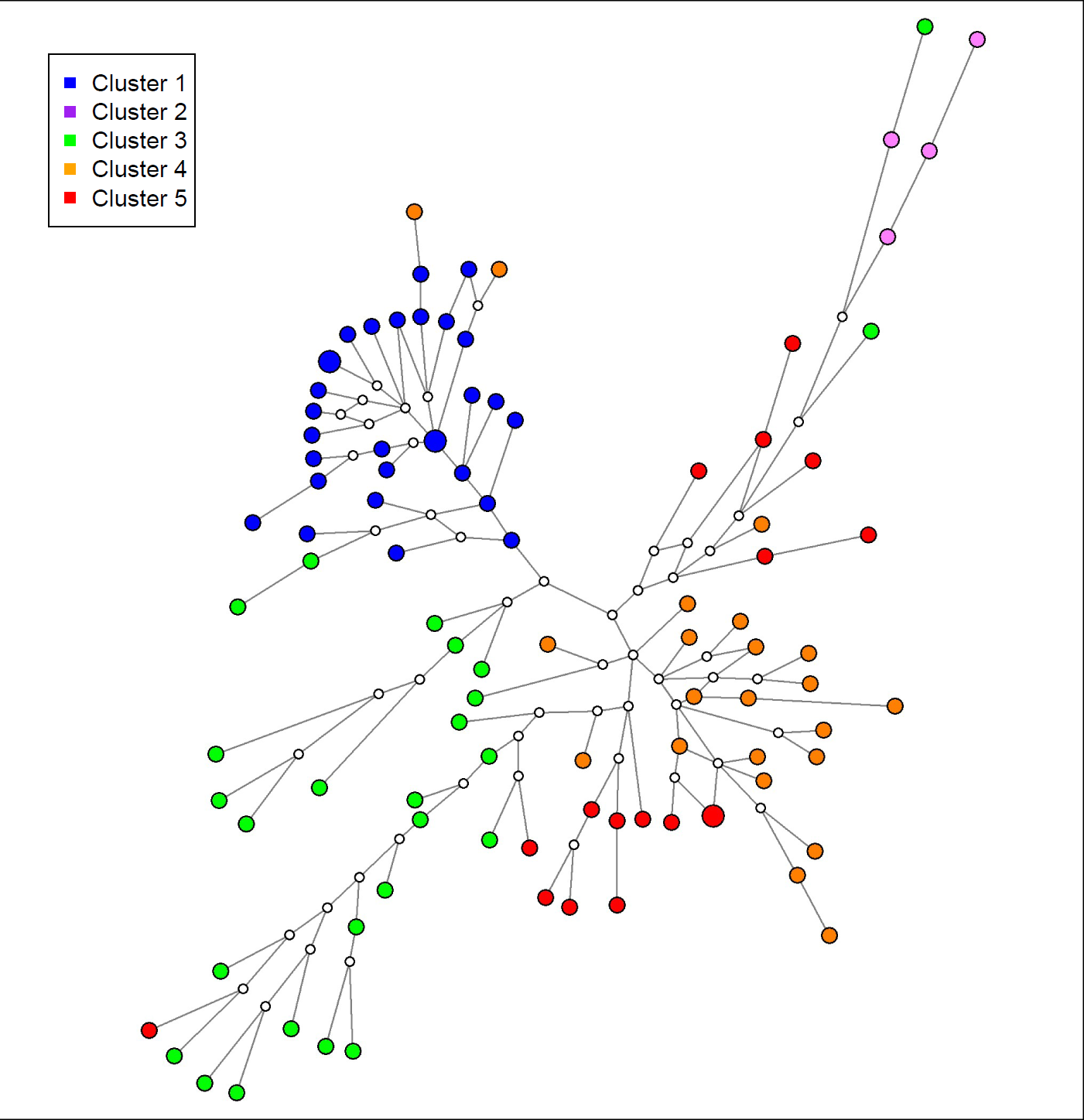

Supplement: Figure S6 — Median joining network for Italian G2a-P15 haplotypes. Individuals have been assigned and colored according to the correspondent DAPC-based clusters (Figure 2). (TIF) [file pone.0065441.s006.tif]

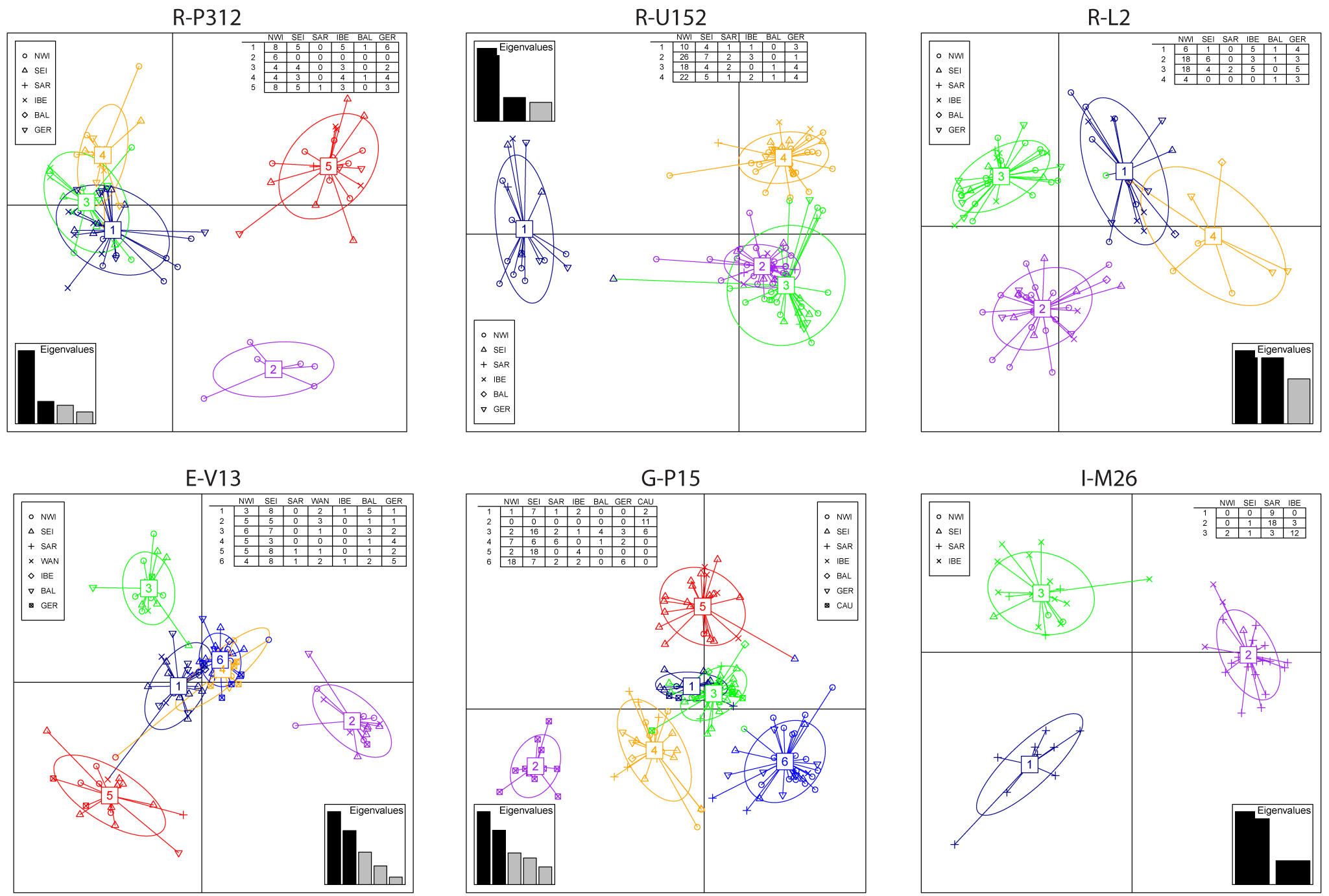

Supplement: Figure S7 — DAPC analysis of STRs variation for the most frequent Y-chromosome haplogroups. Results are based on Italian data and additional comparison samples (NWI; SEI; SAR; IBE: Iberian Peninsula; BAL: Balkan Peninsula; GER: Central-Europe (Germany); CAU: Caucasus; WAN: Western Anatolia; symbols in the legend of each plot). For each plot, the number of different haplotypes per cluster and their geographical distribution are shown in the enclosed table. The DAPC eigenvalues are depicted in the enclosed barplot. (TIF) [file pone.0065441.s007.tif]

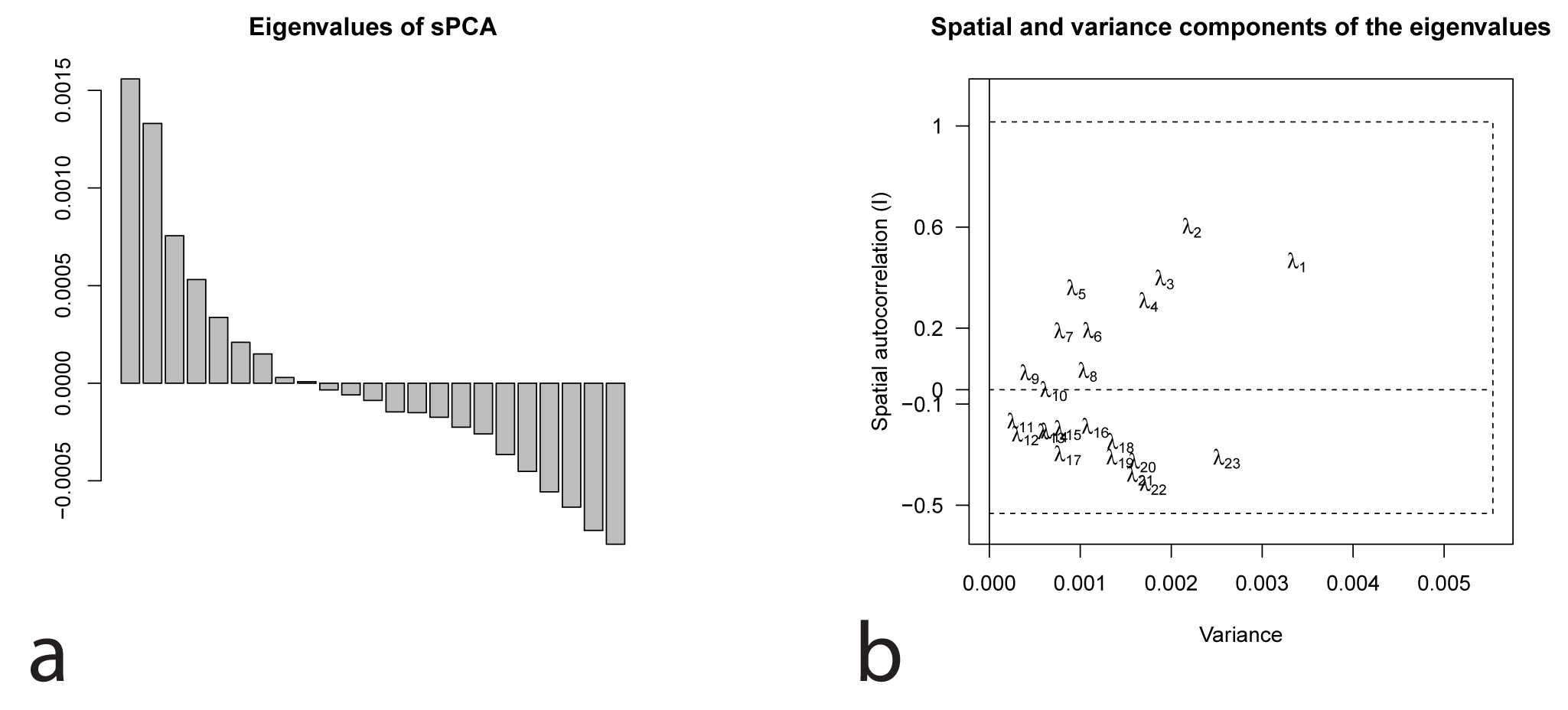

Supplement: Figure S8 — Eigenvalues of mtDNA-based sPCA analysis (A) with their decomposition in spatial and variance components (B). Eigenvalues are obtained maximizing the product of variance and spatial autocorrelation (Moran's I index), and are both positive and negative, depending from Moran's I positive or negative values. Large positive components correspond to global structures; large negative components correspond to local structures (marked genetic differentiation among neighbours). (TIF) [file pone.0065441.s008.tif]

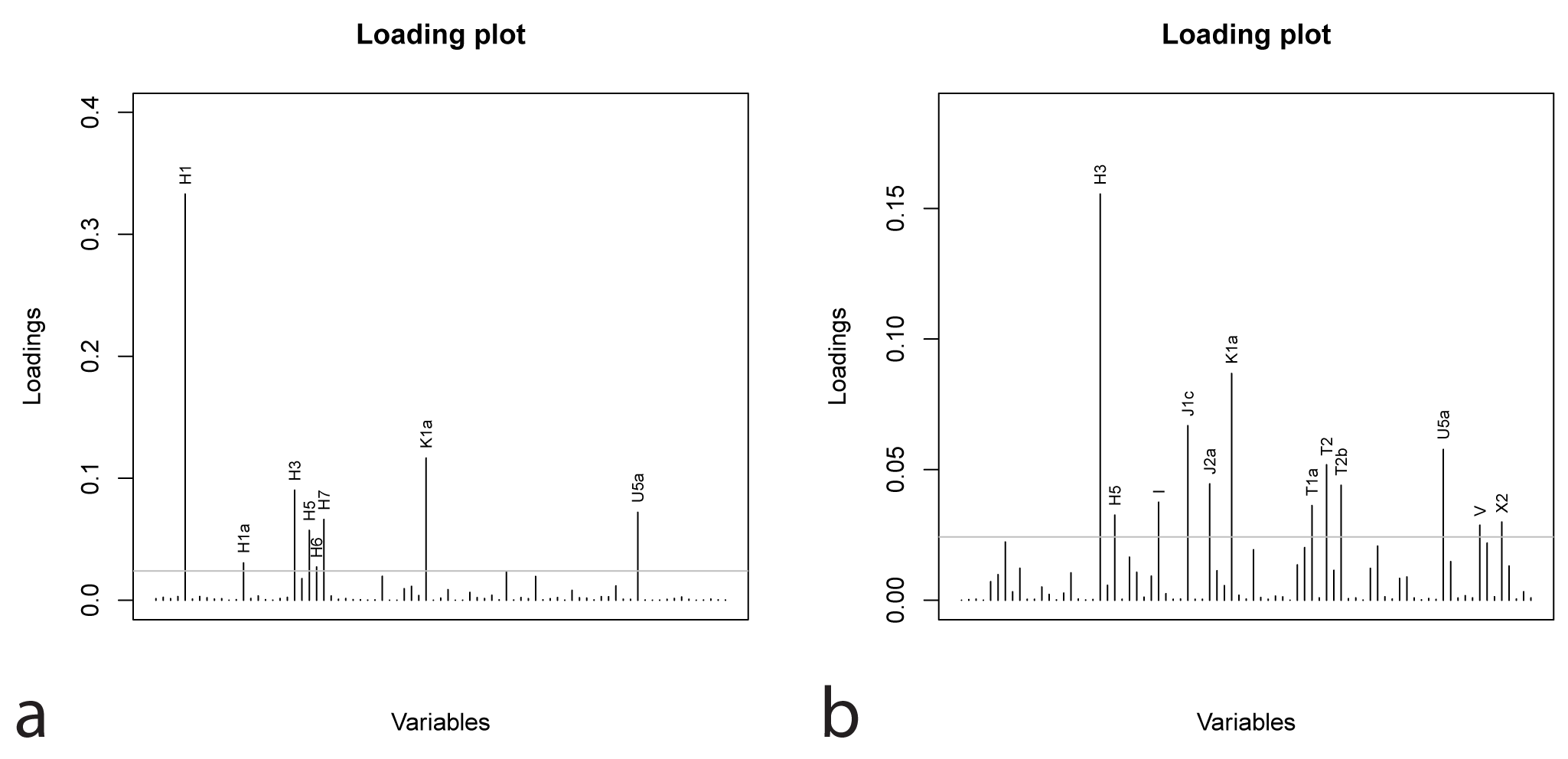

Supplement: Figure S9 — Loadings of the most informative components (a: sPC1, b: sPC2). These values identify mtDNA haplogroups that mostly influence the genetic structure of Italian populations. (TIF) [file pone.0065441.s009.tif]

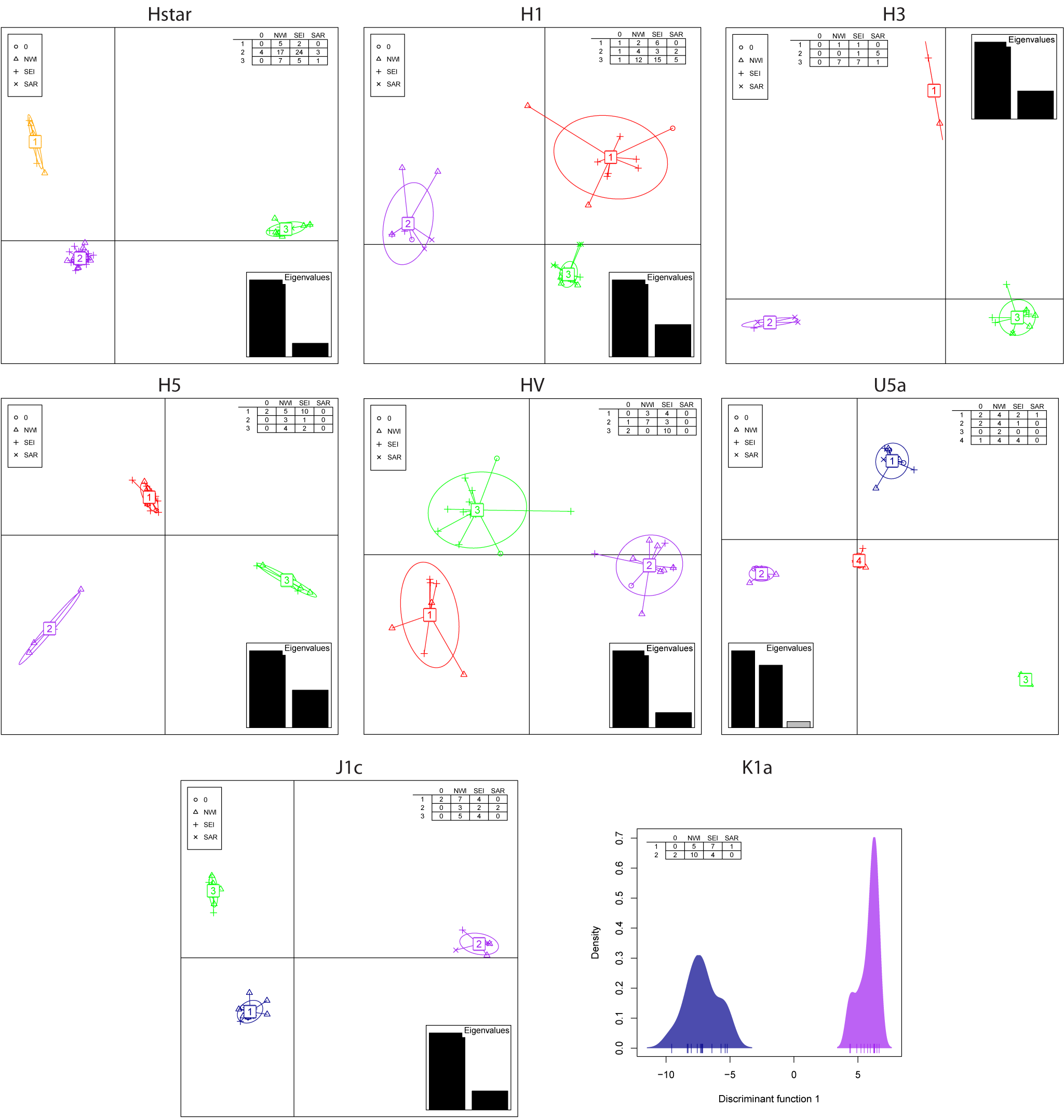

Supplement: Figure S10 — DAPC analysis of HVS variation for the most frequent mtDNA haplogroups (H*, H1, H3, H5, HV, J1c, K1a, U5a) in the Italian data set. Results have been grouped geographically using the same categories as for Y-Chromosome (NWI; SEI; SAR); “0” codes were attributed to those populations for which Y-chromosome information was not available and whose geographical position lies along the boundary between NWI and SEI (Aviano, Terni). For each plot, the number of different haplotypes per cluster and their geographical distribution are shown in the enclosed table. The DAPC eigenvalues are depicted in the enclosed barplot. Haplogroup K1a, including two clusters only, is represented by a single discriminant function (no eigenvalues barplot). (TIF) [file pone.0065441.s010.tif]

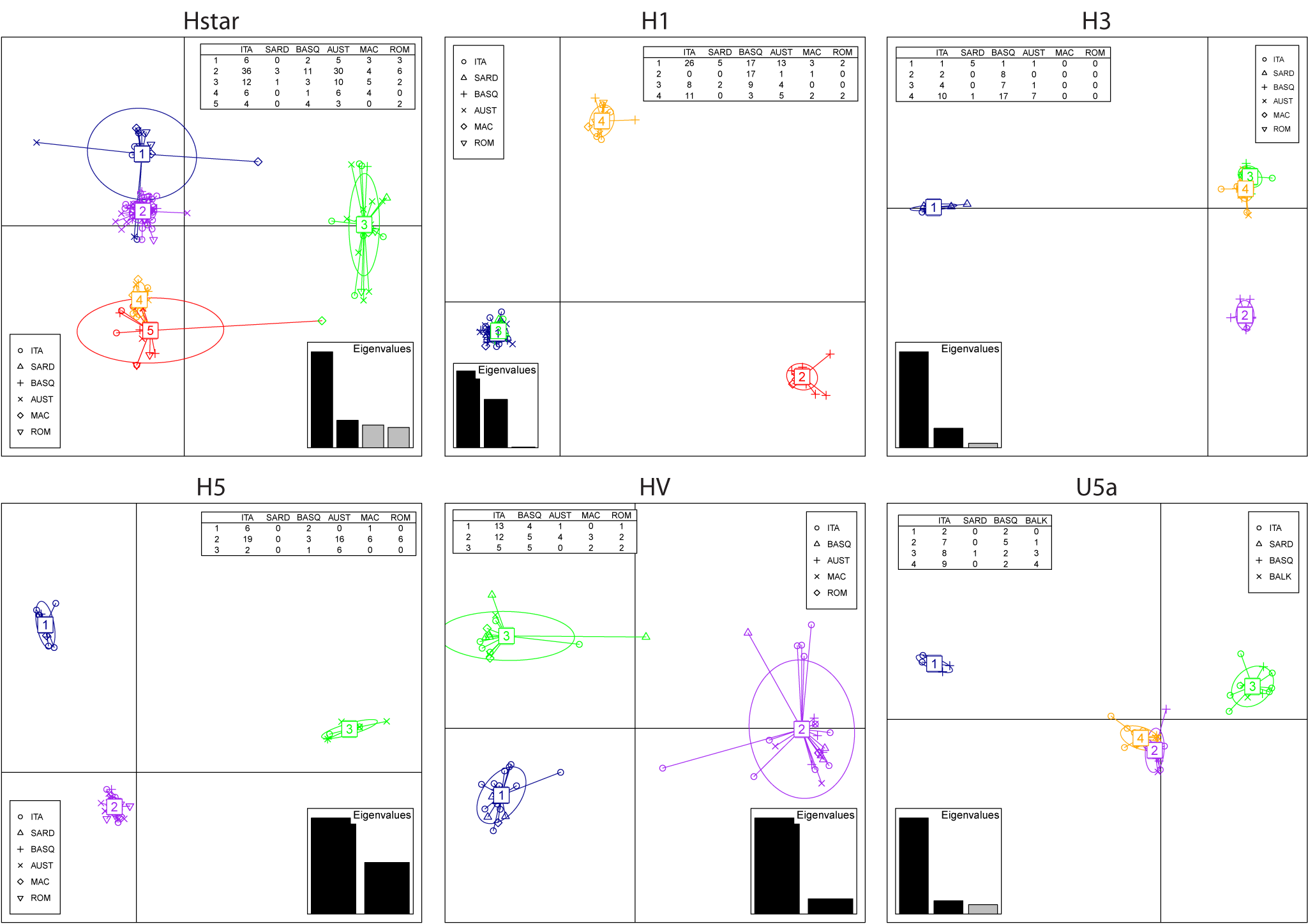

Supplement: Figure S11 — DAPC analysis of HVS variation for the most frequent mtDNAhaplogroups. Results are based on Italian data and comparison European populations (ITA: Continental Italy; SAR: Sardinia; BASQ: Iberian Peninsula (Basques); AUST: Central Europe (Austria); MAC: Macedonians; ROM: Romanians; BALK: Balkan Peninsula; symbols in the legend of each plot). For each plot, the number of different haplotypes per cluster and their geographical distribution are shown in the enclosed table. The DAPC eigenvalues are depicted in the enclosed barplot. (TIF) [file pone.0065441.s011.tif]
