# Supplementary material for: Uniparental Markers in Italy Reveal a Sex-Biased Genetic Structure and Different Historical Strata
Source: PLoS One. 2013 May 29;8(5):e65441. doi: 10.1371/journal.pone.0065441 (PMC3666984; doi:10.1371/journal.pone.0065441)
Supplement: Methods S1 — Spatial Principal Component Analysis (sPCA). Discriminant Analysis of Principal Components. Batwing analysis. “Jackknife-like” procedure for outliers identification. (DOC) [file pone.0065441.s021.doc]

**SUPPLEMENTARY METHODS**

**Spatial Principal Component Analysis (sPCA)**

In order to investigate the spatial distribution of genetic variability within the Italian Peninsula, a spatial principal component analysis (sPCA) was performed on haplogroup frequencies for both Y-Chromosome and Mitochondrial DNA data. Differently from classic PCA, where eigenvalues are calculated by maximizing variance of the data, in sPCA eigenvalues are obtained maximizing the product of variance and spatial autocorrelation (Moran's I index). In order to include spatial information in the analysis, we used a weighting procedure based on a Delaunay connection network [1]. Eigenvalues found by sPCA are both positive and negative, depending from Moran's I positive or negative values. The most informative components are those identified by eigenvalues with the highest absolute values. Large positive components correspond to global structures (cline-like structures); large negative components correspond to local structures (marked genetic differentiation among neighbours). The presence of global or local structures is further assessed by using the Global and Local random test as implemented in the *adegenet* package [2]-[4]. Loadings of the most informative components were used to identify haplogroups that mostly influence the genetic structure of Italian populations.

**Discriminant Analysis of Principal Components (DAPC)**

The genetic variability of mtDNA and Y-chromosome haplotypes within main haplogroups was explored by means of a DAPC analysis. The DAPC method [5] is aimed to describe the diversity between pre-defined groups of observations. Being designed to investigate individual genetic data, the method can be easily adapted to the study of haplotypes within haplogroups. Preliminarily, data are grouped using k-means, a clustering algorithm which finds a given number of clusters maximizing the variation between groups. The algorithm runs on a transformation of the raw data using Principal Component Analysis (PCA). We retained all the principal components in order to conserve all the variation in the original data. The optimal number of clusters is identified by running k-means with increasing values of k (up to a maximum, in our case, of 20). Clustering solutions for different k values are compared calculating Bayesian Information Criterion (BIC). The 'best' solution corresponds to the lowest BIC. The actual DAPC procedure consists of two further steps. First, original data (STR haplotypes) are transformed (centred, in our case) and submitted to a PCA. Second, the retained PCs are passed to a Linear Discriminant Analysis based on the groups identified during the preliminary k-means clustering step. As a result, discriminant functions are constructed as linear combinations of the original variables which have the largest between-group variance and the smallest within-group variance. Membership probabilities are based on the retained discriminant functions. Concerning the first step, it is important to observe that retaining too many PCs with respect to the number of populations can lead to over-fitting the discriminant functions, meaning that membership probabilities may become drastically inflated for the best-fitting cluster, resulting in apparent perfect discrimination. As a consequence, we decided to retain as much PCs are needed to represent ~80% of the variation in the original data. The same problem would hold also for the second step, e.g. the number of retained discriminant functions. In our case, given that the number of investigated clusters is relatively low, all the discriminant functions were retained.

**Batwing analysis**

We established prior distributions covering an expected range congruent with human population history. For mutation rate priors, muprior, these were set to $k=1.47$ and $\theta=2173$ for 25 year generations, where the form of the gamma distribution was $f\left(x;k,\theta\right)dx=\frac{1}{\Gamma\left(k\right)}\left(\frac{x}{\theta}\right)^{k-1}e^{-x/\theta}d\left(\frac{x}{\theta}\right)$. The prior for the ancestral population size was designed to be very flat over the range of likely ancestral values, with $k=1$, and $\theta=0.0001$. The population growth rate priors, alpha prior and betaprior, were set to $k=2$, $\theta=400$, and $k=2$, $\theta=1$. The number of times parameters were updated between samples was Nbetsamp=10, and the number of times trees were changed before updating parameters was treebetN=20. The number of samples between writing the outfile was picgap=1500000. The total number of samples accumulated in the out file was 3.5 million, and 1 million were excluded as burn-in.

SNP information was integrated for the phylogenetic reconstruction, but it was not considered for posterior estimates. Chain convergence was evaluated by running three independent runs (starting from different seeds) and estimating the Gelman and Geweke diagnostic statistics [6], [7] for the parameters of interest with the R package CODA [4], [8].

**“Jackknife-like” procedure for outliers identification**

Being the SD-based time estimation of DAPC clusters sensitively affected by the presence of outliers, a jackknife-like procedure for their identification has been designed as follows. For each DAPC cluster of N individuals, the variance-based estimate (SD) was recomputed N times on a set of N-1 haplotypes, leaving out one individual at a time from the original data set. If one of the N estimates, recalculated with the exclusion procedure, is significantly different from the others, we can suspect the presence of an outlier in the original dataset. In this case the best estimation of time will be the one for which the "outlier" haplotype has been excluded. Otherwise, if none of the recomputed estimates differs significantly compared to the others, we can exclude the presence of outliers. In that case, we retain the time estimate calculated on the whole original dataset. The identification of outlier estimates was performed with Grubbs’ test [9] using the R software *Outliers* package [10].

**References**

1. Brassel KE, Reif D. (1979) A procedure to generate Thiessen polygons. Geogr Anal 325:31-36.
2. Jombart T (2008) adegenet: a R package for the multivariate analysis of genetic markers. Bioinformatics 24: 1403-1405.
3. Jombart T, Devillard S, Dufour AB, Pontier D (2008) Revealing cryptic spatial patterns in genetic variability by a new multivariate method. Heredity 101: 92-103.
4. R Development Core Team (2008) R: A language and environment for statistical computing. Vienna: R Foundation for Statistical Computing. ISBN 3-900051-07-0, URL [http://www.R-project.org](http://www.R-project.org/).
5. Jombart T, Devillard S, Balloux F (2010) Discriminant analysis of principal components: a new method for the analysis of genetically structured populations. BMC Genet. 11:94.
6. Gelman A, Rubin DB (1992) Inference from Iterative Simulation Using Multiple Sequences. Stat Sci 7:457-472.
7. Geweke J (1992) Evaluating the accuracy of sampling-based approaches to calculating posterior moments. In: Bayesian Statistics 4. Oxford (UK): Clarendon Press.
8. Plummer M, Best N, Cowles K, Vines K (2006) CODA: convergence diagnosis and output analysis for MCMC. R News 6:7-11.
9. Grubbs FE (1950) Sample Criteria for testing outlying observations. Ann Math Stat 21:27-58.
10. Komsta L (2006) Processing data for outliers. R News: 6:10-13.
